# Oral Health Care Out-of-Pocket Costs and Financial Hardship: A Scoping Review

**DOI:** 10.1177/00220345241253191

**Published:** 2024-10-17

**Authors:** D. Proaño, H. Huang, S. Allin, B.M. Essue, S. Singhal, C. Quiñonez

**Affiliations:** 1 Faculty of Dentistry, University of Toronto, Toronto, ON, Canada; 2 Institute of Health Policy, Management and Evaluation, Dalla Lana School of Public Health, University of Toronto, Toronto, ON, Canada; 3 North American Observatory on Health Systems and Policies, University of Toronto, Toronto, ON, Canada; 4 Public Health Ontario, Toronto, ON, Canada; 5 Schulich School of Medicine & Dentistry, University of Western Ontario, London, ON, Canada

**Keywords:** health expenditures, dental health services, universal health insurance, health economics, health policy, direct service cost

## Abstract

The objective of this study is to characterize how financial hardship related to oral health care (OHC) out-of-pocket (OOP) spending has been conceptualized, defined, and measured in the literature and to identify evidence gaps in this area. This scoping review follows Arksey and O’Malley’s framework and synthesizes financial hardship from OHC concepts, methodologies, and evidence gaps. We searched Ovid-Medline, Ovid-Embase, PubMed, Web of Science, Scopus, EconLit, Business Source Premier, and the Cochrane Library. Gray literature was sourced from institutional websites (World Health Organization, United Nations, World Bank Group, Organisation for Economic Co-operation and Development, and governmental health agencies) as well as ProQuest Dissertations and Thesis Global. We used defined inclusion and exclusion criteria to select studies published between 2000 and 2023. Of the 1,876 records, 65 met our criteria. The studies conceptualized financial hardship as catastrophic spending, impoverishment, negative coping strategies, bankruptcy, financial burden, food insecurity, and personal financial hardship experience. We found heterogeneity in defining OHC OOP payments and services. Also, financial hardship was frequently measured as catastrophic health expenditure using cross-sectional designs and national household spending surveys from high-income and to a lesser extent lower-middle-income countries. We identify and discuss challenges in terms of conceptualizing financial hardship, study designs, and measurement instruments in the OHC context. Some of the common evidence gaps identified include studying the causal relationship in financial hardship from OHC, assessing the financial hardship and unmet dental needs due to cost relationship, and distinguishing the effect between pain/discomfort and esthetic/cosmetic dental treatments on financial hardship. Financial hardship in OHC needs further exploration and the use of consistent definitions as well must distinguish between treatments alleviating pain/discomfort from esthetic/cosmetic treatments. Our study is relevant for policy makers and researchers aiming to monitor financial protection of OOP payments on OHC in the wake of universal health coverage for oral health.

## Introduction

The United Nations (UN) and World Health Organization (WHO) have recognized the importance of financially protecting people using oral health care (OHC) services to better meet global OHC needs (UN 2019; WHO 2022). Financial protection goals of universal health coverage (UHC) may be hindered by out-of-pocket (OOP) spending on OHC services by placing an undue financial hardship on those using these services (UN 2019). As such, the WHO’s Global Oral Health Action Plan has highlighted the relevance of monitoring the impact of OOP spending on OHC to reach UHC for oral health by 2030 while ensuring financial protection from OHC OOP costs (WHO 2023).

Paying OOP for OHC is a common way in which individuals finance dental treatment, especially since OHC is often excluded from statutory health care packages (Petersen et al. 2020; Winkelmann, Gómez Rossi, Schwendicke, et al. 2022). OOP payments occur for uncovered/uninsured OHC services as direct payments and as co-sharing requirements (i.e., copayments, co-insurance) or informal (“under-the-table”) payments for covered/insured services (Rice et al. 2018; Thomson et al. 2019). Prepayment mechanisms (i.e., taxes, contributions, premiums) or third-party reimbursements for OOP payments are excluded from the conceptualization of OOP spending as these do not occur at the time of receiving treatment (Rice et al. 2018; Thomson et al. 2019). With or without health insurance provisions, some individuals or households must use their own resources to obtain OHC unless they choose to forgo OHC and, as a consequence, potentially worsen their oral health (Thomson et al. 2024).

Recent evidence shows that financial hardship associated with OOP payments for OHC occurs across health care systems of low-, middle-, and high-income countries (Bernabé et al. 2017; Thomson et al. 2019). The WHO Regional Office for Europe determined OOP payments for OHC to be a major driver of “catastrophic health spending” in the European region from 2011 to 2016 (Thomson et al. 2019). Catastrophic health spending is a form of financial hardship in which the household’s “ability to maintain its customary standard of living” is endangered (Berki 1986). Similarly, multi- and single-country studies from low- and middle-income countries show that OOP payments for OHC are associated with catastrophic spending (Masood et al. 2015; Bernabé et al. 2017; Proaño Falconi and Bernabé 2018).

To appropriately monitor UHC’s financial protection goals for OHC, conceptual and methodological challenges in ascertaining financial hardship associated with OOP costs on OHC need to be addressed (Allison et al. 2023; WHO 2023). Moreover, the OHC context warrants special attention as it is commonly neglected by statutory packages, which places a higher burden on the patient’s side by paying OOP (Petersen et al. 2020; Winkelmann, Gómez Rossi, Schwendicke, et al. 2022). Consequently, there is a need to differentiate between treatments to resolve pain/discomfort versus esthetic/cosmetic treatment in affecting financial hardship, as the vision of the global strategy on oral health focuses on achieving UHC for preventive, restorative, and rehabilitation services related to oral diseases and conditions (WHO 2023). Some methodological challenges on this topic are related to the different approaches used to capture the impact of OOP spending for OHC (Organisation for Economic Co-operation and Development [OECD] 2021; Winkelmann, Gómez Rossi, and van Ginneken 2022), such as adapting catastrophic spending measures to OHC (Masood et al. 2015; Sun et al. 2016). In addition, there is no consensus among researchers or institutions on the best-suited threshold for catastrophic spending measures, and the ones currently used (10% and 25%) to meet sustainable development goals in financial protection have been criticized for equity concerns (Thomson et al. 2019). Further, there is a major knowledge gap in health economics and economic monitoring of OHC expenditure in oral health (Watt et al. 2020).

Given conceptual and methodological gaps in this area, this study seeks to characterize the concepts, definitions, and measurement of financial hardship in OHC OOP costs. As such, the main research question of our review is: How has financial hardship related to OHC OOP spending been conceptualized, defined, and measured in the literature? Further, subquestions include the following: (1) What are the key characteristics of the conceptual frameworks used to study financial hardship related to paying OOP for OHC? (2) What are the key definitions used in studying financial hardship related to paying OOP for OHC? (3) What are the key characteristics of the methodological approaches and challenges in studying financial hardship related to paying OOP for OHC? (4) What evidence gaps exist in the literature on the experience of financial hardship related to paying OOP for OHC?

## Methods

A scoping review methodology following Arksey and O’Malley’s (2005) framework was conducted to characterize concepts, definitions, and methodological approaches used in the literature on experiencing financial hardship associated with dental OOP payments. Compared with a systematic review, a scoping review allows us to make use of the broad literature to clarify concepts and identify evidence gaps (Munn et al. 2018).

### Search Strategy

We followed the Preferred Reporting Items for Scoping Reviews (Appendix File 1). Studies were accessed through the published and gray literature between January 2000 and December 2022. Peer-reviewed articles were screened from Ovid-Medline, Ovid-Embase, PubMed, Web of Science, Scopus, EconLit, Business Source Premier, and the Cochrane Library (see search strategies in Appendix File 2). To guide our search, we used the Participant Concept Context framework, in which the participant is a household/individual, the concept is financial hardship, and the context is OOP payments for OHC. A manual search was performed on the literature referenced within the included studies. Gray literature was included by screening institutional websites from the WHO, UN, World Bank Group, OECD, and English-speaking national health agencies (Australia, Canada, Ireland, New Zealand, United States, and the United Kingdom), as well as theses/dissertations through ProQuest Dissertations and Thesis Global. A working protocol was developed but not registered and is available upon request.

### Study Selection

All retrieved sources were imported into Covidence, where duplicate studies were removed, and a guided screening was performed. We included a broad range of sources (editorials, book chapters, reports, guidelines, policy documents, expert opinions, case studies, and studies with a quantitative, qualitative, or mixed-methods approach) in which financial hardship was reported as a consequence or associated with OOP payments for OHC, costs were estimated using the patient’s perspective, the full text was in English, publication occurred between January 2000 and December 2023, and availability was through the University of Toronto Libraries. We excluded sources in which it was not possible to distinguish if financial hardship was related to OHC OOP payments, the authors did not report on the measurement of financial hardship from OHC, OOP payments were known to be reimbursed, or the source was a conference abstract or retracted study.

**Figure 1.**
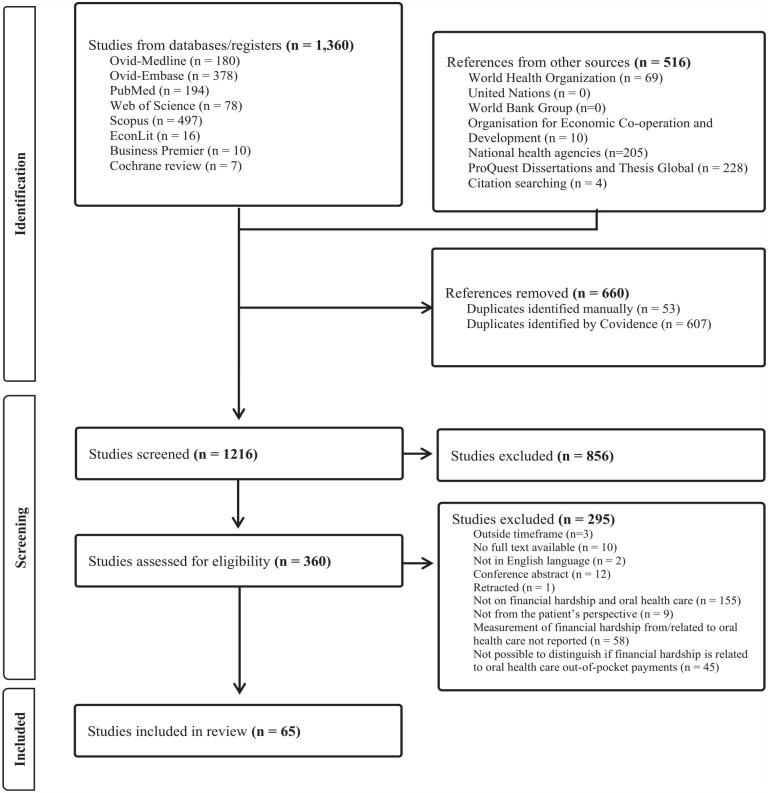
Prisma diagram.

We recorded the number of selected studies by source and the reasons for exclusion using a Prisma diagram (Page et al. 2021), shown in Figure 1. To enhance consistency in our screening of studies, we conducted a pilot test among 25 random studies in which 2 independent reviewers (D.P. and H.H.) reached 81% agreement corrected for chance using the Kappa statistic. Disagreements that emerged were resolved through consensus among the reviewers.

### Charting the Data

Covidence software was used for data charting on the selected studies and to ensure consistency in the extraction of all relevant study features. The relevant fields charted were study characteristics, concept(s) and conceptual framework(s), definition(s), and measurement of financial hardship in OHC. We also included the challenges reported by researchers in terms of measuring financial hardship from OHC as well as evidence gaps.

### Collating, Summarizing, and Reporting the Results

The Levac et al. (2010) framework guided our analysis as we produced a numerical summary of the study characteristics, performed a descriptive qualitative content analysis to identify overarching themes corresponding to our research objectives, and discussed our findings in terms of their meaning to future research and policy implications. Our summary of the study characteristics included the country income groups covered based on the 2023 World Bank Group classification. Furthermore, key characteristics of the conceptual frameworks, methodological approaches, and methodological challenges were recorded and strengths and weaknesses were discussed. Evidence gaps were also identified. In the discussion of our findings, we present a conceptual framework based on the conceptual relationships found in financial hardship related to OOP spending on OHC.

## Results

### Study Characteristics

**Figure 2.**
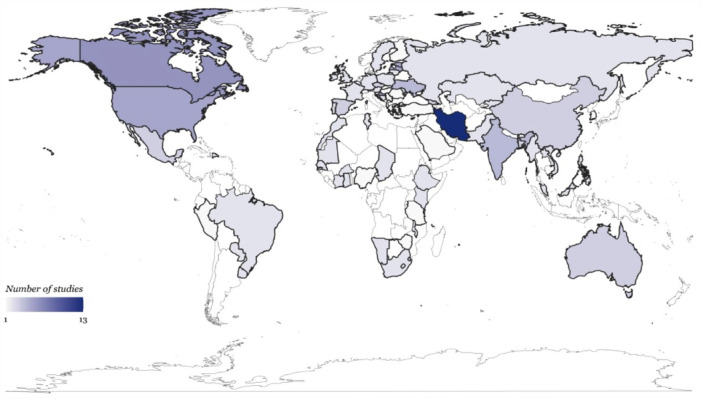
Geographical coverage from studies on financial hardship related to oral health care out-of-pocket spending. All countries studied within a multicountry study are included in the map, so the number of countries covered (75) is greater than the number of studies included in our review (65).

We found 1,876 records, 65 of which met the criteria for inclusion in the review (Fig. 1). A full list of the studies can be found in Appendix Table 1. In Figure 2, we provide the geographical country coverage of the studies reviewed. Furthermore, we have summarized the study characteristics based on the financial hardship concepts and measures identified in our review and defined in Table 1. In general, reviewed studies were mostly from high and lower-middle-income countries, cross-sectional in nature, used secondary data, analyzed national household surveys, and reported on catastrophic health expenditure (Table 2).

**Table 1.** Financial Hardship Concepts, Measures, and Explanations in the Oral Health Care Context.

Concept	Measure	Explanation
1. Catastrophic spending	Calculation of catastrophic health expenditure	Exceeding a prespecified fraction of the household’s finances on OOP spending for health care services (OHC included).
	Calculation of catastrophic dental health expenditure	Exceeding a prespecified fraction of the household’s finances on OOP spending for OHC.
2. Impoverishment	Calculation of impoverishment	Paying for OHC pushes household under the poverty line.
3. Financial burden	Self-reported financial burden from OHC	Participant identifies that OHC OOP payments are burdensome.
4. Negative coping strategies	Identification of distress financing financial coping strategies	Financial coping strategy to pay for OHC OOP spending generates financial hardship.
5. Bankruptcy	Identification of bankruptcy	Using external sources (such as a debt agency) to identify participants who have experienced bankruptcy and then relating this to OHC OOP spending.
6. Food insecurity	Self-reported food insecurity	Participant identifies themselves as experiencing food insecurity and then relating this to OHC OOP spending.
7. Personal experience	Description of personal financial hardship experience from paying OOP for OHC	Participant recalls and describes how paying for OHC OOP was a financial hardship.

OHC, oral health care; OOP, out-of-pocket.

**Table 2.** Summary of Study Characteristics by Financial Hardship Measures.

Measure/Characteristic		*n* ^a^
1. Catastrophic spending		
1.1 Catastrophic health expenditure (*n* = 43)		
Country income group^b^	High income	24
	Upper-middle income	3
	Lower-middle income	15
	High and upper-and-lower middle income	1
	Upper-and-lower middle income and low income	1
Data collection	Primary data collection	9
	Secondary data collection	34
Study design	Systematic review	1
	Cross-sectional	42
Instrument^c^	National household spending survey	30
	Health care spending survey	2
	Household health survey	10
1.2 Catastrophic dental health expenditure (*n* = 10)		
Country income group^b^	High income	4
	Upper-middle income	2
	Lower-middle income	3
	Upper-and-lower middle income and low income	1
Data collection	Primary data collection	3
	Secondary data collection	7
Study design	Cross-sectional	10
Instruments	Author-created questionnaire	2
	National household spending survey	3
	National oral health survey	2
	Household health survey	1
	Dental charts	1
	Unclear	1
2. Impoverishment (*n* = 1)		
Country income group^b^	Upper-and-lower middle income and low income	1
Data collection	Secondary data collection	1
Study design	Cross-sectional	1
Instruments	Household health survey	1
3. Self-reported financial burden from oral health care (*n* = 6)		
Country income group^b^	High income	3
	Lower-middle income	2
	High and upper-middle income	1
Data collection	Primary data collection	4
	Secondary data collection	2
Study design	Cross-sectional	6
Instruments	Author-created questionnaire	2
	Household survey	1
	National oral health survey	2
	Unclear	1
4. Distress financing (*n* = 1)		
Country income group^b^	Lower-middle income	1
Data collection	Primary data collection	1
Study design	Cross-sectional	1
Instrument	Author-created questionnaire	1
5. Bankruptcy (*n* = 1)		
Country income group^b^	High income	1
Data collection	Primary data collection	1
Study design	Cross-sectional	1
Instrument	Insolvency files and author-created questionnaire	1
6. Self-reported food insecurity (*n* = 3)		
Country income group^b^	High-income	3
Data collection	Primary data collection	1
	Secondary data collection	2
Study design	Cross-sectional	3
Instruments	National health care spending survey and food insecurity questionnaire	2
	Author-created questionnaire and food insecurity questionnaire	1
7. Financial hardship experience from paying OOP for OHC (*n* = 2)		
Country income group^b^	High income	2
Data collection	Primary data collection	2
Study design	Qualitative design	2
Instruments	Narrative inquiry	1
	Focus group	1

Two studies used more than 1 financial hardship measure, so the total number of studies shown (67) is greater than the number of studies included in our review (65). OOP, out-of-pocket; OHC, oral health care.

a See full list of references in Appendix Table 2.

b From the 2023 World Bank Group country income group list.

c Excludes systematic review.

### Conceptual Frameworks

The studies reviewed did not provide any specific conceptual framework tailored to financial hardship in the OHC context. Nevertheless, some studies described relevant relationships affecting financial hardship from OHC. For instance, the WHO Regional Office for Europe infers a correlation between countries with relatively strong financial protection and OHC as a source of financial hardship (Thomson et al. 2019). Where financial protection is weak (i.e., low breadth, depth, and scope of OHC coverage), OHC is still a source of financial hardship for high-income households but not for low-income households who generally choose to skip dental visits and face greater unmet dental needs (Thomson et al. 2019). In addition, financial hardship related to OHC seems to relate to the household’s ability to pay rather than the population’s oral health needs (Pérez-Núñez et al. 2007).

### Definitions

We found variations in how studies defined financial hardship (Table 1), although some of these concepts share similarities (Appendix Table 3). Studies also varied in how they defined OOP payments, what types of dental services were consumed, and the recall period used for dental OOP spending (Appendix Table 4). Twenty-one studies defined OOP payments as including payments for uncovered/uninsured services (i.e., direct payments) and covered/insured services (i.e., co-payments/deductibles; informal “under-the-table” payments), excluding prepayment and third-party reimbursement (e.g., governmental or private insurance). Other definitions of OOP spending included only direct payments or in combination with cost-sharing but did not indicate any prepayment exclusion.

Fifty-seven studies defined OHC broadly without specifying which services were consumed (see the full list of OHC services covered in Appendix Table 4). Generally, only studies reporting on catastrophic dental health expenditure and self-reported financial burden were more specific. Three studies included medicines prescribed by dentists as part of the OOP spending on OHC, and 1 included dental inpatient services.

Twenty-two studies were unclear on the OHC utilization timeframe. Seventeen studies analyzing national household spending (or budget) surveys, health care spending surveys, and national dental surveys generally used a 1-y recall period. Alternatively, a 1-mo recall period was frequent among the studies using a household health survey (e.g., World Health Survey questionnaire). Other recall periods used ranged from 6 mo to 3 y (Appendix Table 4).

### Key Characteristics of Methodological Approaches

Table 3 describes the distinct methodological approaches used to measure financial hardship in the OHC context. Studies quantifying OHC OOP spending and the household’s finances (i.e., income or consumption) measured catastrophic health expenditure or its “dental” variation (catastrophic dental health expenditure) and impoverishment. The catastrophic spending studies assessed if the household’s health care (or OHC) OOP spending exceeds a fraction of the household’s income or consumption (i.e., budget share) or the household’s effective income after subsistence needs are deducted (i.e., capacity to pay). The study measuring impoverishment, instead, calculated the change in the number of households experiencing poverty after deducting health care (including OHC) OOP spending from their finances.

**Table 3.** Methodologies to Assess Financial Hardship Used in the Oral Health Care Context.

Measure/Method	Approach to Analysis
1.1 Catastrophic health expenditure (CHE)	
a. Budget share (household’s finances defined as)	
• Consumption (*n* = 3) ➢ CHE thresholds: 10%,* 25%, 40%	(i) Proportion of CHE and any OHC OOP spending (ii) Share of OHC OOP spending from health care OOP spending among CHE cases
• Income (*n* = 2) ➢ CHE thresholds: 5%, 10%*	(i) Proportion of CHE and any OHC OOP spending
b.1 Capacity to pay (household finances = consumption minus)	
• Partial normative food spending^a^ (*n* = 14) ➢ CHE thresholds: 25%, 40%*	(i) Proportion of CHE and any OHC OOP spending (ii) Share of OHC OOP spending from health care OOP spending among CHE cases (iii) Probability of CHE and any OHC OOP spending
• Normative food, housing, and utilities spending^b^ (*n* = 21) ➢ CHE threshold: 40%	(i) Share of OHC OOP spending from health care OOP spending among CHE cases (ii) Correlation between proportion of households with CHE and OHC OOP to total OOP spending ratio
• Total food spending^c^ (*n* = 2) ➢ CHE threshold: 40%	(i) Proportion of CHE and any OHC OOP spending (ii) Probability of CHE and any OHC OOP spending
b.2 Capacity to pay (household finances = income minus)	
• Partial normative food spending^a^ (*n* = 2) ➢ CHE threshold: 40%	(i) Proportion of CHE and any OHC OOP spending (ii) Probability of CHE and any OHC OOP spending
1.2 Catastrophic dental health expenditure (CDHE)	
a. Budget share (household’s finances defined as)	
• Consumption (*n* = 1) ➢ CDHE threshold: 20%	(i) Proportion of CDHE
• Income (*n* = 7) ➢ CDHE thresholds: 10%, 20%, 40%	(i) Proportion of CDHE
b. Capacity to pay (household finances = consumption minus)	
• Total food spending^b^ (*n* = 1) ➢ CDHE thresholds: 30%	(i) Proportion of CDHE
• Partial normative food spending^a^ (*n* = 1) ➢ CDHE threshold: 40%	(i) Proportion of CDHE (ii) Average extent exceeded from the catastrophic threshold (overshoot) (iii) Mean overshoot among CDHE cases
2. Impoverishment (IMPOV)	
• Household finances = consumption (*n* = 1) ➢ Poverty line: 44th and 55th ranked household food consumption average from the sample	(i) Proportion of IMPOV and any OHC OOP spending (ii) Probability of IMPOV and OHC OOP spending
3. Self-reported financial burden from oral health care	
• “In the past 3 years has the cost of dental care been a financial burden to you?” (*n* = 1) ➢ Indicator: “Yes”	(i) Proportion of self-reported financial burden from OHC
• “In the last 12 months, how much of a financial burden have dental visits been for you?” (*n* = 2) ➢ Indicator: “A large burden”	(i) Proportion of self-reported financial burden from OHC
• “Does your dental expenditure affect other day to day expenditure?” (*n* = 1) ➢ Indicator: “Yes”	(i) Proportion of self-reported financial burden from OHC
• “Do you feel dental expenditure is really a burden to your family?” (*n* = 1) ➢ Indicator: “Yes”	(i) Proportion of self-reported financial burden from OHC
• “To what extent were the costs of dental examinations or treatments a financial burden to your household during the past 12 months?” (*n* = 1) ➢ Indicator: “A heavy burden” or “somewhat a burden”	(i) Proportion of self-reported financial burden from OHC
• Financial impact on dental caries treatment^d^ (n = 1) ➢ Indicator: “Serious impact” or “very serious impact”	(i) Proportion of self-reported financial burden from OHC
4. Distress financing	
• Type of financing households used to pay for health care^d^ (*n* = 1) ➢ Indicator (any of the following): loans, selling assets, extra working hours, ex gratia payment by other household member, reducing food spending, removing children from school	(i) Proportion of distress financing for OHC OOP spending
5. Bankruptcy	
• Unable to keep up with their debts and have either to sell all of their assets or reach an agreement with their debtors (*n* = 1) ➢ Indicator: Insolvency files	(i) Proportion of participants experiencing bankruptcy and spending a “large amount” OOP on OHC
6. Self-reported food insecurity	
• Presence of food insecurity issues (8 or 10 questions)^e^ (*n* = 2) ➢ Indicator: “Yes” to any food insecurity issue	(i) Proportion of participants self-reporting food insecurity and any OHC OOP spending (ii) Probability of experiencing food insecurity and any OHC OOP spending
• Frequency of food insecurity issues (3 questions) in terms of being worried there would be enough to eat in the last year, did not have enough to eat, or did not eat the desired quality or variety of food because of lack of money^f^ (*n* = 1) ➢ Indicator: “Often” or “sometimes” to any food insecurity issue	
7. Financial hardship experience from paying out-of-pocket for oral health care	
• In-depth interview on the “sequential costs” (i.e., risking paying rent) due to OHC OOP spending (*n* = 1)	(i) Thematic analysis
• Focus group on accessing OHC (*n* = 1)	(ii) Narrative analysis

See the full list of references in Appendix Table 5. OOP, out-of-pocket; OHC, oral health care.

* Indicates most frequent threshold used among reviewed studies.

a Calculated by averaging the 44^th^- and 55th-ranked household food consumption from the sample (among those with negative capacity to pay the actual food spending is employed).

b Calculated by averaging the food, rent, and utilities (water, electricity, gas and other fuels used for cooking and heating) spending from the 25th- and 35th-ranked household consumption (equivalized to household size and composition) from the sample (those with a negative capacity to pay are considered catastrophic).

c Calculated from the household’s total food consumption.

d Exact phrasing of the question is unavailable.

e From the 2016/17 Medical Expenditure Panel Survey in the United States.

^f^From the 2003 Canadian Community Health Survey.

The other studies used different methodologies. Financial burden questionnaires (mostly author created) were used to ask participants to identify if they experienced a (large) financial burden due to OHC; validation processes were not disclosed among these studies. The distress financing study used a validated questionnaire in face-to-face interviews to identify households resorting to distress financing to pay for dental OOP. The study on bankruptcy used insolvency files to identify adults experiencing bankruptcy and mailed them a self-administered questionnaire to assess if they had a large dental OOP expenditure (>5,000 Canadian dollars). Food insecurity questionnaires combined with health care spending surveys or author-created questionnaires were used to assess the relationship between food insecurity and dental OOP spending. Finally, financial hardship was also reported by participants who expressed financial hardship experiences after dental OOP spending.

### Key Characteristics of Methodological Challenges

Measurement challenges faced by researchers vary on the methodological approach used to measure financial hardship in OHC (Appendix Table 6). In catastrophic spending methodologies, the conceptual challenges described pointed to the inability to address nonspenders as they may have experienced financial barriers and forgone OHC. Moreover, instrument challenges were focused on the recall period of OOP spending and the lack of specificity in terms of dental services paid for and study design challenges addressed the cross-sectional design of these studies.

In terms of the self-reported financial burden, some of the challenges pointed to the lack of information on the amount of dental OOP spending being made by each household member. In addition, since the questionnaires used commonly contain close-ended questions, participants are unable to expand on why they self-identified OOP spending in OHC as a financial burden (Appendix Table 6).

The other studies also reported methodological challenges. The measurement of distress financing lacked the inclusion of indirect costs (i.e., productivity losses), as in such cases the participant’s reporting was considered inaccurate. The study on bankruptcy found a very low response rate among those experiencing bankruptcy, which may systematically affect those in a worse financial situation. Researchers employing food insecurity questionnaires mention the lack of information on the participant’s contextual characteristics (i.e., living conditions or neighborhood characteristics), the different definitions of what food insecurity entails for different organizations, and the difficulty in establishing causal relationships. Studies using a qualitative assessment identified the lack of reporting on the participant’s income as a main methodological challenge (Appendix Table 6).

### Evidence Gaps

In terms of the evidence gaps, we noted some studies mentioning the inability to measure causality in financial hardship from dental OOP costs (Appendix Table 7). We also found gaps in terms of the relationship between catastrophic spending and unmet dental needs due to cost, the effect of health policy interventions, the longitudinal effect of financial hardship, the impact of specific coping mechanisms, and the specific dental treatment driving financial hardship.

We found 2 other evidence gaps coming from the self-reported financial burden from OHC studies and the qualitative studies on financial hardship from OHC. One study described a gap in assessing the structure of financial hardship and the causal relationship of financial hardship from OHC and analyzing the contextual characteristics (i.e., socioeconomic characteristics, type and health insurance coverage, and health system characteristics; Vojvodic et al. 2022). The other study recognized gaps in assessing the determinants of financial hardship, the extent to which experiencing varying levels of financial hardship affects the hardships that families face, and measuring the effects using a societal perspective (Peck and Segal 2006; Appendix Table 7).

## Discussion

Our scoping review focused on how financial hardship associated with OHC OOP spending had been conceptualized, defined, and measured, which is relevant as OHC is often excluded from statutory packages. Further, there is a need to differentiate between emergency treatments to alleviate oral pain and discomfort to more comprehensive treatments including endodontics and prosthesis to cosmetic treatments such as veneers or orthodontics for esthetic reasons, which matters for achieving UHC OHC goals (Quiñonez and Vujicic 2020; Benzian et al. 2022). As such, our review will help to appropriately measure financial protection goals in achieving UHC for oral health (Allison et al. 2023; WHO 2023). Although we did not find a unified definition of financial hardship in the OHC context, most studies conceptualize and measure catastrophic health expenditure as financial hardship, which allows for the comparison of the impact of OOP costs in OHC to other health care services.

### Concepts and Definitions

**Figure 3.**
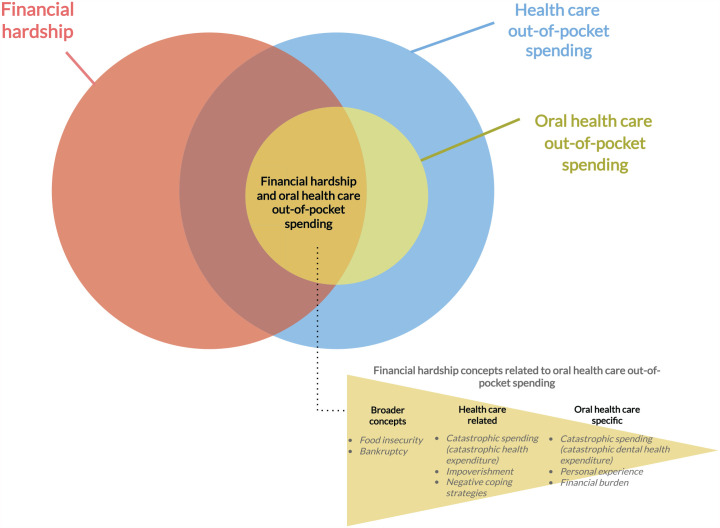
Conceptual framework of financial hardship related to oral health care out-of-pocket spending.

We have constructed a conceptual framework portraying the relationship between financial hardship (in a broader sense), health care OOP spending, and OHC OOP spending, as well as the concepts used to assess financial hardship related to OHC OOP spending (see Fig. 3). As shown, some financial hardship concepts related to OHC OOP spending also relate to a broader sense of financial hardship (i.e., food insecurity and bankruptcy), which is affected by health care and non–health care OOP costs. Other measures are more specific to health care OOP spending, which may include OHC OOP spending (i.e., catastrophic health expenditure and impoverishment). Catastrophic dental health expenditure, financial hardship experienced from OHC OOP costs, and self-reported financial burden are concepts bound to the OOP spending in OHC. In other words, these more OHC-specific financial hardship concepts do not allow for any other health care OOP spending. Although our study identified 7 different financial hardship concepts, we did not find other concepts regularly used in the health economics and health care literature (e.g., financial toxicity, financial stress, affordability) to be measuring the financial impact of OOP costs in OHC. In general, however, the financial hardship concepts found in our study do align with how the field of financial risk protection frames their research studies (Bhatia et al. 2022; Rahman et al. 2022) and with the WHO framework on financial hardship from OOP spending on health care (WHO and International Bank for Reconstruction and Development/The World Bank 2021).

Although our framework organizes the information from the studies reviewed, we identified some areas that need more conceptual clarity. For example, most of the studies reviewed did not provide a clear definition of the type of OHC services that are paid OOP. As such, the use of an international standard definition of OOP spending would be desirable, such as defining OOP in accordance with the System of Health Accounts 2011 (OECD et al. 2017) and OHC services as per the 2018 Classification of Individual Consumption According to Purpose (UN Department of Economic and Social Affairs Statistics Division 2018). Nonetheless, researchers are often limited to the information collected from household spending surveys, which often do not collect information on the structure and financing of OOP payments (O’Donnell 2019; Thomson et al. 2019).

In household spending (or budget) surveys, the use of OHC services is broad and limits researchers in identifying what types of OHC service(s) drive financial hardship (Bernabé et al. 2017). From a policy and equity perspective, it matters to know if it is to attend to immediate pain/infection (emergency), avoid future oral health problems (preventive), for rehabilitative care (prosthesis), and/or for purely esthetic/cosmetic treatments that are driving financial hardship. Importantly, OHC OOP spending may be perceived differently for varying types of procedures by sociodemographic attributes. Modifications to household budget surveys should consider that increasing the number of items on OHC services may increase spending estimates (Heijink et al. 2011), whereas decomposing and disaggregating services into finer subclasses may alternatively yield lower OOP spending estimates because it requires households to recall the specific name(s) of the service(s) provided (Agorinya et al. 2021). Other sources of OHC OOP spending may be sought, such as using health surveys and dental records (Rannan-Eliya and Lorenzoni 2010).

### Measurements and Challenges

Researchers have commonly estimated the OOP spending on OHC contribution/relationship to catastrophic health expenditure. However, none of these analyses measure if OOP spending on OHC was necessary for a household to experience catastrophic spending or calculated the extent to which households exceed the catastrophic threshold because of OOP spending on OHC.

In catastrophic dental health expenditure studies, an important challenge is the lack of a suitable catastrophic threshold, as the thresholds commonly used in catastrophic health expenditure studies consider a range of OOP spending for health care services and not solely for OHC. Moreover, finding a suitable threshold for OHC risks incomparable findings to other catastrophic health expenditure studies and OOP spending for health care services.

Self-reported financial burden questionnaires lack proper analysis of their measurement properties, such as addressing content validity, reliability, criterion validity, and responsiveness (De Vet et al. 2011). Analyzing measurement properties are context and population specific (De Vet et al. 2011), let alone the fact that self-reported measures reflect only changes to current living standards and potentially not desired and anticipated living standards (Brüggen et al. 2017).

There is a lack of literature on other financial hardship methodologies, which does not allow us to generalize on methodological challenges. We found only 1 study conducted for distress financing, 1 for bankruptcy, 2 with qualitative assessments, and 3 on food insecurity. Nevertheless, from these studies, we can point to the lack of information and granularity on household finances and the potential importance of understanding contextual characteristics from a measurement perspective.

### Evidence Gaps

Based on the studies reviewed, there are several evidence gaps in the literature. Some of these gaps are directly related to the limitations and challenges of the financial hardship concept (e.g., households with cost barriers showing no catastrophic expenditure), the study design (e.g., lack of longitudinal designs), and the instruments employed (e.g., household spending surveys). Moreover, the lack of longitudinal methodologies limits researchers in understanding how OHC OOP payments affect the continuation of care or how OHC treatment for one household member affects the care for other members. Furthermore, no studies analyzed whether households change consumption patterns and affect living standards by paying OOP for OHC.

### Limitations

There are some study weaknesses to consider. First, we did not assess the quality of these studies or the quality of the measurement tools proposed, as these are not within scope. Second, our study evaluated only studies published in English and may have missed relevant literature from non–English-speaking countries. Nonetheless, 70 of the 74 countries covered in our review were nonnative English-speaking countries. Third, our eligibility criteria excluded studies published before the year 2000. Although there were more studies published before our year of publication exclusion, slightly more than 90% of the studies on UHC have been published since the year 2000 (Ghanbari et al. 2021). In this case, even if we missed some studies, we believe we still have significant coverage of the literature on financial hardship in the OHC context. Also, it is likely that older studies may not be relevant or have strong implications for today’s health care and political context. Fourth, by focusing only on the financial hardship from OOP costs on OHC, we have left out unmet/forgone OHC and other relevant OHC barriers (e.g., geographical barriers, cultural barriers, waiting times) that may adversely affect people needing OHC services.

### Policy and Research Implications

This scoping review provides policy makers and researchers with valuable information on financial hardship concepts and measurement in the OHC context. Specifically, this review is of value to policy makers in understanding the different tools used to monitor financial protection in OOP costs on OHC. For this monitoring, we make a call to responsible agencies of household spending surveys to consider modifying their questionnaire items on OOP spending on OHC services to capture spending from pain/discomfort treatments and esthetic/cosmetic treatments or at least to separate purely esthetic/cosmetic OOP payments from overall OHC spending. Further research on financial hardship in OHC needs to address the impact of different health/OHC interventions, the effect of OHC OOP spending on catastrophic health expenditure, better distinguishing what types of OHC services (i.e., treatments to resolve pain/discomfort vs. esthetic/cosmetic treatments) drive financial hardship, and understanding how the different types of OOP spending structures in OHC affect financial hardship in distinct OHC systems.

## Conclusion

This scoping review summarized the financial hardship literature in the OHC context; appraised the concepts, definitions, methodologies, and evidence gaps in this field; and provided recommendations for researchers. Our work is relevant to researchers and policy makers aiming to monitor financial protection goals relevant to UHC for oral health by 2030. From our review, we have identified financial hardship in OHC to be defined and measured commonly as catastrophic spending, but this is an area that requires further exploration and use of consistent definitions as well as understanding what types of dental treatments drive financial hardship.

## Author Contributions

D. Proaño, contributed to conception, design, data acquisition, analysis, and interpretation, drafted and critically revised the manuscript; H. Huang, contributed to data acquisition, analysis, and interpretation, critically revised the manuscript; All these authors contributed to “conception and design, critically revised the manuscript.” In other words, C. Quiñonez also contributed to the design of the study (as shows the original manuscript). All authors gave final approval and agree to be accountable for all aspects of the work.

## Supplemental Material

sj-docx-1-jdr-10.1177_00220345241253191 – Supplemental material for Oral Health Care Out-of-Pocket Costs and Financial Hardship: A Scoping Review Supplemental material, sj-docx-1-jdr-10.1177_00220345241253191 for Oral Health Care Out-of-Pocket Costs and Financial Hardship: A Scoping Review by D. Proaño, H. Huang, S. Allin, B.M. Essue, S. Singhal and C. Quiñonez in Journal of Dental Research
