# Supplementary material for: Oral Health Care Out-of-Pocket Costs and Financial Hardship: A Scoping Review
Source: J Dent Res. 2024 Oct 17;103(12):1197–208. doi: 10.1177/00220345241253191 (PMC11562291; doi:10.1177/00220345241253191)
Supplement: sj-docx-1-jdr-10.1177_00220345241253191 – Supplemental material for Oral Health Care Out-of-Pocket Costs and Financial Hardship: A Scoping Review [file sj-docx-1-jdr-10.1177_00220345241253191.docx]

**Oral Health Care Out-of-Pocket Costs & Financial Hardship: A Scoping Review**

D. Proaño^1^, H. Huang^1^, S. Allin^2,3^, B.M. Essue^2^, S. Singhal^1,4^, and C. Quiñonez^1,5^

1. Faculty of Dentistry, University of Toronto, Toronto, Ontario, Canada.

2. Institute of Health Policy, Management and Evaluation, Dalla Lana School of Public Health, University of Toronto, Toronto, ON, Canada

3. North American Observatory on Health Systems and Policies, University of Toronto, Toronto, ON, Canada

4. Public Health Ontario, Toronto, Ontario, Canada.

5. Schulich School of Medicine & Dentistry, University of Western Ontario, London, Ontario, Canada.

**Appendix File 1: PRISMA** (Tricco AC, Lillie E, Zarin W, O'Brien KK, Colquhoun H, Levac D, et al. PRISMA Extension for Scoping Reviews (PRISMAScR): Checklist and Explanation. Ann Intern Med. 2018;169:467–473. doi: 10.7326/M18-0850.)

**Preferred Reporting Items for Systematic reviews and Meta-Analyses extension for Scoping Reviews (PRISMA-ScR) Checklist**

| **SECTION** | **ITEM** | **PRISMA-ScR CHECKLIST ITEM** | **REPORTED ON PAGE #** |
| --- | --- | --- | --- |
| **TITLE** | | | |
| Title | 1 | Identify the report as a scoping review. | 1 |
| **ABSTRACT** | | | |
| Structured summary | 2 | Provide a structured summary that includes (as applicable): background, objectives, eligibility criteria, sources of evidence, charting methods, results, and conclusions that relate to the review questions and objectives. | 2 |
| **INTRODUCTION** | | | |
| Rationale | 3 | Describe the rationale for the review in the context of what is already known. Explain why the review questions/objectives lend themselves to a scoping review approach. | 3 |
| Objectives | 4 | Provide an explicit statement of the questions and objectives being addressed with reference to their key elements (e.g., population or participants, concepts, and context) or other relevant key elements used to conceptualize the review questions and/or objectives. | 4 |
| **METHODS** | | | |
| Protocol and registration | 5 | Indicate whether a review protocol exists; state if and where it can be accessed (e.g., a Web address); and if available, provide registration information, including the registration number. | 4 |
| Eligibility criteria | 6 | Specify characteristics of the sources of evidence used as eligibility criteria (e.g., years considered, language, and publication status), and provide a rationale. | 4 |
| Information sources* | 7 | Describe all information sources in the search (e.g., databases with dates of coverage and contact with authors to identify additional sources), as well as the date the most recent search was executed. | 4 |
| Search | 8 | Present the full electronic search strategy for at least 1 database, including any limits used, such that it could be repeated. | Appendix file 2 |
| Selection of sources of evidence† | 9 | State the process for selecting sources of evidence (i.e., screening and eligibility) included in the scoping review. | 4,5 |
| Data charting process‡ | 10 | Describe the methods of charting data from the included sources of evidence (e.g., calibrated forms or forms that have been tested by the team before their use, and whether data charting was done independently or in duplicate) and any processes for obtaining and confirming data from investigators. | 5 |
| Data items | 11 | List and define all variables for which data were sought and any assumptions and simplifications made. | 5 |
| Critical appraisal of individual sources of evidence§ | 12 | If done, provide a rationale for conducting a critical appraisal of included sources of evidence; describe the methods used and how this information was used in any data synthesis (if appropriate). | NA |
| Synthesis of results | 13 | Describe the methods of handling and summarizing the data that were charted. | 5 |
| **RESULTS** | | | |
| Selection of sources of evidence | 14 | Give numbers of sources of evidence screened, assessed for eligibility, and included in the review, with reasons for exclusions at each stage, ideally using a flow diagram. | 6 |
| Characteristics of sources of evidence | 15 | For each source of evidence, present characteristics for which data were charted and provide the citations. | Appendix Table 2 |
| Critical appraisal within sources of evidence | 16 | If done, present data on critical appraisal of included sources of evidence (see item 12). | NA |
| Results of individual sources of evidence | 17 | For each included source of evidence, present the relevant data that were charted that relate to the review questions and objectives. | 7-10, 13,14 & Appendix Table 3-7 |
| Synthesis of results | 18 | Summarize and/or present the charting results as they relate to the review questions and objectives. | 11-12, 15 |
| **DISCUSSION** | | | |
| Summary of evidence | 19 | Summarize the main results (including an overview of concepts, themes, and types of evidence available), link to the review questions and objectives, and consider the relevance to key groups. | 15-17 |
| Limitations | 20 | Discuss the limitations of the scoping review process. | 17,18 |
| Conclusions | 21 | Provide a general interpretation of the results with respect to the review questions and objectives, as well as potential implications and/or next steps. | 18 |
| **FUNDING** | | | |
| Funding | 22 | Describe sources of funding for the included sources of evidence, as well as sources of funding for the scoping review. Describe the role of the funders of the scoping review. | 18 |

JBI = Joanna Briggs Institute; PRISMA-ScR = Preferred Reporting Items for Systematic reviews and Meta-Analyses extension for Scoping Reviews.

* Where *sources of evidence* (see second footnote) are compiled from, such as bibliographic databases, social media platforms, and Web sites.

† A more inclusive/heterogeneous term used to account for the different types of evidence or data sources (e.g., quantitative and/or qualitative research, expert opinion, and policy documents) that may be eligible in a scoping review as opposed to only studies. This is not to be confused with *information sources* (see first footnote).

‡ The frameworks by Arksey and O’Malley (6) and Levac and colleagues (7) and the JBI guidance (4, 5) refer to the process of data extraction in a scoping review as data charting*.*

§ The process of systematically examining research evidence to assess its validity, results, and relevance before using it to inform a decision. This term is used for items 12 and 19 instead of "risk of bias" (which is more applicable to systematic reviews of interventions) to include and acknowledge the various sources of evidence that may be used in a scoping review (e.g., quantitative and/or qualitative research, expert opinion, and policy document).

**Appendix File 2:** Search strategies employed for Ovid-Medline, Ovid-Embase, PubMed, Web of Science, Scopus, EconLit, Business Source Premier, and the Cochrane Library and grey literature. Search conducted in June 2023 (October 2023 for grey literature) and updated in February 2024.

**Ovid- Medline**

| **#** | **Searches** | **Results** |
| --- | --- | --- |
| 1 | Human/ or family/ or exp nuclear family/ or persons/ or caregivers/ or (famil* or household* or person* or adult* or caregiver*).tw,kf. [Participant] | 22922405 |
| 2 | Financial stress/ or ((financial or economic) adj3 (risk* or protection* or hardship* or burden* or toxicit* or pressure* or difficult* or vulnerab* or stress* or distress* or challeng* or strain* or worr* or problem* or shock* or barrier* or well-being or wel?being or satisfaction*)).mp. | 64959 |
| 3 | (catastroph* adj3 (spend* or expen*)).mp. | 1355 |
| 4 | (bankruptcy or debt* or impoverish* or poverty).mp. | 84907 |
| 5 | (cost* adj1 (barrier or high or large or excessive)).mp. | 31996 |
| 6 | (afford* or unafford*).mp. | 139802 |
| 7 | exp Dental care/ or dental health services/ or ((dental or oral or stomato*) adj2 (care or service*)).tw,kf. or (dent* or tooth or teeth).tw,kf. | 550754 |
| 8 | exp Health expenditures/ or (health adj2 (expenditure* or spending)).mp. or out-of-pocket.mp. or out of pocket.mp. or (out adj4 pocket).mp. or OOP.mp. or cost shar*.mp. or cost-shar*.mp. or co-pay*.mp. or copay*.mp. or (user adj (fee or charge)).mp. | 44975 |
| 9 | 2 or 3 or 4 or 5 or 6 [Concept] | 311277 |
| 10 | 7 and 8 [Context] | 1021 |
| 11 | 1 and 9 and 10 [PCC] | 157 |
| 12 | limit 11 to (english language and yr="2000 - 2023") | 180 |

**Ovid- Embase**

| **#** | **Searches** | **Results** |
| --- | --- | --- |
| 1 | Human/ or family/ or exp nuclear family/ or persons/ or caregivers/ or (famil* or household* or person* or adult* or caregiver*).tw,kf. [Participant] | 28726334 |
| 2 | Financial stress/ or ((financial or economic) adj3 (risk* or protection* or hardship* or burden* or toxicit* or pressure* or difficult* or vulnerab* or stress* or distress* or challeng* or strain* or worr* or problem* or shock* or barrier* or well-being or wel?being or satisfaction*)).mp. | 92496 |
| 3 | (catastroph* adj3 (spend* or expen*)).mp. | 1544 |
| 4 | (bankruptcy or debt* or impoverish* or poverty).mp. | 90106 |
| 5 | (cost* adj1 (barrier or high or large or excessive)).mp. | 42743 |
| 6 | (afford* or unafford*).mp. | 179473 |
| 7 | exp Dental care/ or dental health services/ or ((dental or oral or stomato*) adj2 (care or service*)).tw,kf. or (dent* or tooth or teeth).tw,kf. | 655999 |
| 8 | exp Health expenditures/ or (health adj2 (expenditure* or spending)).mp. or out-of-pocket.mp. or out of pocket.mp. or (out adj4 pocket).mp. or OOP.mp. or cost shar*.mp. or cost-shar*.mp. or co-pay*.mp. or copay*.mp. or (user adj (fee or charge)).mp. | 368335 |
| 9 | 2 or 3 or 4 or 5 or 6 [Concept] | 208786 |
| 10 | 7 and 8 [Context] | 2964 |
| 11 | 1 and 9 and 10 [PCC] | 416 |
| 12 | limit 11 to (english language and yr="2000 - 2023") | 378 |

**Pubmed**

("family"[MeSH Terms] OR "persons"[MeSH Terms] OR "caregivers"[MeSH Terms] OR "famil*"[Text Word] OR "person*"[Text Word] OR "caregiver*"[Text Word] OR "household*"[Text Word] OR "adult*"[Text Word]) AND ("financial risk*"[All Fields] OR "financial protection*"[All Fields] OR "financial hardship*"[All Fields] OR "financial burden*"[All Fields] OR "financial toxi*"[All Fields] OR "financial stress*"[All Fields] OR "financial pressure*"[All Fields] OR "financial difficult*"[All Fields] OR "financial vulner*"[All Fields] OR "financial distress*"[All Fields] OR "financial challenge*"[All Fields] OR "financial strain*"[All Fields] OR "financial worr*"[All Fields] OR "financial problem*"[All Fields] OR "financial shock*"[All Fields] OR "financial barrier*"[All Fields] OR "financial satisfaction" OR "financial well-being"[All Fields] OR "financial wellbeing"[All Fields] OR "financial health"[All Fields] OR "economic risk*"[All Fields] OR "economic protection*"[All Fields] OR "economic hardship*"[All Fields] OR "economic toxic*"[All Fields] OR "economic stress*"[All Fields] OR "economic pressure*"[All Fields] OR "economic difficult*"[All Fields] OR "economic vulner*"[All Fields] OR "economic distress*"[All Fields] OR "economic challenge*"[All Fields] OR "economic strain*"[All Fields] OR "economic worr*"[All Fields] OR "economic problem*"[All Fields] OR "economic shock*"[All Fields] OR "economic barrier*"[All Fields] OR "economic satisfaction*"[All Fields] OR "economic well-being"[All Fields] OR "economic health"[All Fields] OR "catastroph*"[All Fields] OR "bankruptcy"[All Fields] OR "debt*"[All Fields] OR "impoverish*"[All Fields] OR "poverty"[All Fields] OR "cost barrier*"[All Fields] OR "high cost*"[All Fields] OR "large cost*"[All Fields] OR "excessive cost*" OR "afford*" OR "unafford*" [All Fields]) AND ("dental care"[Text Word] OR "dental health services"[MeSH Terms] OR "dental care"[Text Word] OR "oral care"[Text Word] OR "dental service*"[Text Word] OR "oral service*"[Text Word] OR "stomato*"[Text Word] OR "dent*"[Text Word] OR "teeth"[Text Word] OR "tooth"[Text Word]) AND ("health expenditures"[MeSH Terms] OR "expenditure*"[All Fields] OR "spending*"[All Fields] OR "out-of-pocket"[All Fields] OR "out-of-pocket"[All Fields] OR "OOP"[All Fields] OR "cost shar*"[All Fields] OR "co pay*"[All Fields] OR "user fee"[All Fields] OR "user charge"[All Fields]) AND 2000/01/01:2023/12/31[Date - Entry] AND "English"[Language]

Results = 194

**Web of Science**

| Search Query |  | Results |
| --- | --- | --- |
| 13 | (#12) AND DOP=(2000-01-01/2023-12-31) | 78 |
| 12 | #11 AND LA=(English) | 78 |
| 11 | #1 AND #7 AND #10 | 81 |
| 10 | #8 AND #9 | 365 |
| 9 | (((((((((ALL=("health expenditure*")) OR ALL=("expenditure*) OR ALL=("spending*) OR ALL=("out-of-pocket")) OR ALL=("out of pocket")) OR ALL=("OOP")) OR ALL=("cost shar*")) OR ALL=("cost-shar*")) OR ALL=("co-pay*")) OR ALL=("co pay*")) OR ALL=("user fee")) OR ALL=("user charge") | 24693 |
| 8 | (((TS=("dental care")) OR (TS=("dental service*"))) OR (TS=("dental health care")) OR (TS=("dental health service")) OR (TS=("oral care")) OR (TS=("oral service")) OR (TS=(stomato*)) OR (TS=(dent*)) OR (TS=(teeth)) OR (TS=(tooth))) | 539163 |
| 7 | #2 OR #3 OR #4 OR #5 OR #6 | 800123 |
| 6 | (((ALL=("cost barrier*")) OR ALL=("large cost*")) OR ALL=("high cost*")) OR ALL=("excessive cost*") OR ALL=("afford*") OR ALL=("unafford*") | 385734 |
| 5 | (((ALL=(bankruptcy)) OR ALL=(debt*)) OR ALL=(impoverish*)) OR ALL=(poverty) | 238369 |
| 4 | (ALL=(catastroph*)) | 89784 |
| 3 | ALL=("economic risk*") OR ALL=("economic protection*") OR ALL=("economic hardship*") OR ALL=("economic burden*") OR ALL=("economic toxi*") OR ALL=("economic pressure*") OR ALL=("economic difficult*") OR ALL=("economic vulner*") OR ALL=("economic stress*") OR ALL=("economic distress*") OR ALL=("economic challenge*") OR ALL=("economic strain*") OR ALL=("economic worr*") OR ALL=("economic problem*") OR ALL=("economic shock*") OR ALL=("economic barrier*") OR ALL=("economic well-being") OR ALL=("economic wellbeing") OR ALL=("economic satisfaction*") OR ALL=(“economic health”) | 58580 |
| 2 | ALL=("financial risk*") OR ALL=("financial protection*") OR ALL=("financial hardship*") OR ALL=("financial burden*") OR ALL= ("financial toxi*") OR ALL=("financial pressure*") OR ALL=("financial difficult*") OR ALL=("financial vulner*") OR ALL=("financial stress*") OR TS=("financial distress*") OR ALL= ("financial challenge*") OR ALL=("financial strain*") OR ALL=("financial worr*") OR ALL=("financial problem*") OR ALL=("financial shock*") OR ALL=("financial barrier*") OR ALL=("financial well-being") OR ALL=("financial wellbeing") OR ALL=("financial welbeing") OR ALL=("financial satisfaction*") OR ALL=(“financial health”) | 50356 |
| 1 | (((TS=(famil*)) OR TS=(household*)) OR TS=(person*)) OR TS=(caregiver*) OR TS=(adult*) | 5802277 |

**Scopus**

TITLE-ABS-KEY ( famil* OR household* OR person* OR caregiver* OR adult* OR caregiver* ) AND ALL ( {financial risk*} OR {financial protection*} OR {financial hardship*} OR {financial burden*} OR {financial toxicit*} OR {financial pressure*} OR {financial difficult*} OR {financial vulnerabilit*} OR {financial stress*} OR {financial distress*} OR {financial challenge*} OR {financial strain*} OR {financial worr*} OR {financial problem*} OR {financial shock*} OR {financial barrier*} OR {financial well-being} OR {financial satisfaction*} OR {financial wellbeing} OR {financial welbeing} OR {financial health} OR {economic risk*} OR {economic protection*} OR {economic hardship*} OR {economic burden*} OR {economic toxicity*} OR {economic pressure*} OR {economic difficulty*} OR {economic vulnerability*} OR {economic stress*} OR {economic distress*} OR {economic challenge*} OR {economic strain*} OR {economic worr*} OR {economic problem*} OR {economic shock*} OR {economic barrier*} OR {economic satisfaction*} OR {economic well-being} OR {economic wellbeing} OR {economic health} OR catastroph* OR bankruptcy OR debt* OR impoverish* OR poverty OR {cost barrier} OR {high cost} OR {large cost} OR {excessive cost} ) AND TITLE-ABS-KEY ( {dental care} OR {dental service*} OR {dental health care} OR {dental health service} OR dent* OR tooth OR teeth ) AND ( expenditure* OR spending OR {out-of-pocket} OR oop OR {cost shar*} OR {cost-shar*} OR {co-pay*} OR {co pay*} OR {user fee} OR {user charge} ) OR {afford*} OR {unafford*} AND PUBYEAR > 2000 AND PUBYEAR > 2000 AND PUBYEAR < 2024 AND ( LIMIT-TO ( LANGUAGE , "English" ) )

Results = 497

**Econ Lit**

| **#** | **Query** | **Results** |
| --- | --- | --- |
| S5 | (SU ( ( ( ((financial or economic) N2 (risk* or protection* or hardship* or burden* or toxicit* or pressure* or difficult* or vulnerab* or stress* or distress* or challeng* or strain* or worr* or problem* or shock* or barrier* or satisfaction* or well-being or wellbeing or health)) ) ) OR SU ( ( (catastroph* N3(spend* or expen*)) ) ) OR SU ( ( (bankruptcy or debt* or impoverish* or poverty) ) ) OR SU ( ( (cost N1 (barrier or high or large or excessive)) ) ) ) OR AB ( ( ( ((financial or economic) N2 (risk* or protection* or hardship* or burden* or toxicit* or pressure* or difficult* or vulnerab* or stress* or distress* or challeng* or strain* or worr* or problem* or shock* or barrier* or satisfaction* or well-being or wellbeing or health)) ) ) OR ( ( (catastroph* N3(spend* or expen*)) ) ) OR ( ( (bankruptcy or debt* or impoverish* or poverty) ) ) OR ( ( (cost N1 (barrier or high or large or excessive)) ) ) ) OR TI ( ( ( ((financial or economic) N2 (risk* or protection* or hardship* or burden* or toxicit* or pressure* or difficult* or vulnerab* or stress* or distress* or challeng* or strain* or worr* or problem* or shock* or barrier* or satisfaction* or well-being or wellbeing or health)) ) ) OR ( ( (catastroph* N3(spend* or expen*)) ) ) OR ( ( (bankruptcy or debt* or impoverish* or poverty) ) ) OR ( ( (cost N1 (barrier or high or large or excessive)) ) ) )) AND (S1 AND S2 AND S3 AND S4) | 16 |
|  |  |  |
|  |  |  |
| S4 | SU ( ( ( ( ((financial or economic) N2 (risk* or protection* or hardship* or burden* or toxicit* or pressure* or difficult* or vulnerab* or stress* or distress* or challeng* or strain* or worr* or problem* or shock* or barrier* or satisfaction* or well-being or wellbeing or health)) ) ) OR ( ( (catastroph* N3(spend* or expen*)) ) ) OR ( ( (bankruptcy or debt* or impoverish* or poverty or affor* or unafford*) ) ) OR ( ( (cost N1 (barrier or high or large or excessive)) ) ) ) ) OR AB ( ( ( ( ((financial or economic) N2 (risk* or protection* or hardship* or burden* or toxicit* or pressure* or difficult* or vulnerab* or stress* or distress* or challeng* or strain* or worr* or problem* or shock* or barrier* or satisfaction* or well-being or wellbeing or health)) ) ) OR ( ( (catastroph* N3(spend* or expen*)) ) ) OR ( ( (bankruptcy or debt* or impoverish* or poverty or affor* or unafford*) ) ) OR ( ( (cost N1 (barrier or high or large or excessive)) ) ) ) ) OR TI ( ( ( ( ((financial or economic) N2 (risk* or protection* or hardship* or burden* or toxicit* or pressure* or difficult* or vulnerab* or stress* or distress* or challeng* or strain* or worr* or problem* or shock* or barrier* or satisfaction* or well-being or wellbeing or health)) ) ) OR ( ( (catastroph* N3(spend* or expen*)) ) ) OR ( ( (bankruptcy or debt* or impoverish* or poverty or affor* or unafford*) ) ) OR ( ( (cost N1 (barrier or high or large or excessive)) ) ) ) ) | 228310 |
|  |  |  |
|  |  |  |
| S3 | SU ( ( spending* OR expenditure* OR "out-of-pocket" or OOP or cost shar* or co-pay* or (user N1 (fee or charge) ) ) OR AB ( ( spending* OR expenditure* OR "out-of-pocket" or OOP or cost shar* or co-pay* or (user N1 (fee or charge) ) ) OR TI ( ( spending* OR expenditure* OR "out-of-pocket" or OOP or cost shar* or co-pay* or (user N1 (fee or charge) ) ) | 104673 |
| S2 | SU ( dent* OR stomato* OR teeth OR tooth OR ((dental or oral) N2 (care or service*)) OR ) OR AB ( dent* OR stomato* OR teeth OR tooth OR ((dental or oral) N2 (care or service*)) OR ) OR TI ( dent* OR stomato* OR teeth OR tooth OR ((dental or oral) N2 (care or service*)) OR ) | 761 |
| S1 | SU ( ( (famil* or household* or person*or caregiver*) ) ) OR AB ( ( (famil* or household* or person*or caregiver*) ) ) OR TI ( ( (famil* or household* or person*or caregiver*) ) ) | 167400 |

**Business Source Premier,**

Filters: Published between 2000 (Jan) -2022 (December)

| **#** | **Query** | **Results** |
| --- | --- | --- |
| S5 | (SU ( ( ( ((financial or economic) N2 (risk* or protection* or hardship* or burden* or toxicit* or pressure* or difficult* or vulnerab* or stress* or distress* or challeng* or strain* or worr* or problem* or shock* or barrier* or satisfaction* or well-being or wellbeing or health)) ) ) OR SU ( ( (catastroph* N3(spend* or expen*)) ) ) OR SU ( ( (bankruptcy or debt* or impoverish* or poverty) ) ) OR SU ( ( (cost N1 (barrier or high or large or excessive)) ) ) ) OR AB ( ( ( ((financial or economic) N2 (risk* or protection* or hardship* or burden* or toxicit* or pressure* or difficult* or vulnerab* or stress* or distress* or challeng* or strain* or worr* or problem* or shock* or barrier* or satisfaction* or well-being or wellbeing or health)) ) ) OR ( ( (catastroph* N3(spend* or expen*)) ) ) OR ( ( (bankruptcy or debt* or impoverish* or poverty) ) ) OR ( ( (cost N1 (barrier or high or large or excessive)) ) ) ) OR TI ( ( ( ((financial or economic) N2 (risk* or protection* or hardship* or burden* or toxicit* or pressure* or difficult* or vulnerab* or stress* or distress* or challeng* or strain* or worr* or problem* or shock* or barrier* or satisfaction* or well-being or wellbeing or health)) ) ) OR ( ( (catastroph* N3(spend* or expen*)) ) ) OR ( ( (bankruptcy or debt* or impoverish* or poverty) ) ) OR ( ( (cost N1 (barrier or high or large or excessive)) ) ) )) AND (S1 AND S2 AND S3 AND S4) | 10 |
|  |  |  |
|  |  |  |
| S4 | SU ( ( ( ( ((financial or economic) N2 (risk* or protection* or hardship* or burden* or toxicit* or pressure* or difficult* or vulnerab* or stress* or distress* or challeng* or strain* or worr* or problem* or shock* or barrier* or satisfaction* or well-being or wellbeing or health)) ) ) OR ( ( (catastroph* N3(spend* or expen*)) ) ) OR ( ( (bankruptcy or debt* or impoverish* or poverty or affor* or unafford*) ) ) OR ( ( (cost N1 (barrier or high or large or excessive)) ) ) ) ) OR AB ( ( ( ( ((financial or economic) N2 (risk* or protection* or hardship* or burden* or toxicit* or pressure* or difficult* or vulnerab* or stress* or distress* or challeng* or strain* or worr* or problem* or shock* or barrier* or satisfaction* or well-being or wellbeing or health)) ) ) OR ( ( (catastroph* N3(spend* or expen*)) ) ) OR ( ( (bankruptcy or debt* or impoverish* or poverty or affor* or unafford*) ) ) OR ( ( (cost N1 (barrier or high or large or excessive)) ) ) ) ) OR TI ( ( ( ( ((financial or economic) N2 (risk* or protection* or hardship* or burden* or toxicit* or pressure* or difficult* or vulnerab* or stress* or distress* or challeng* or strain* or worr* or problem* or shock* or barrier* or satisfaction* or well-being or wellbeing or health)) ) ) OR ( ( (catastroph* N3(spend* or expen*)) ) ) OR ( ( (bankruptcy or debt* or impoverish* or poverty or affor* or unafford*) ) ) OR ( ( (cost N1 (barrier or high or large or excessive)) ) ) ) ) | 356,807 |
|  |  |  |
|  |  |  |
| S3 | SU ( ( spending* OR expenditure* OR "out-of-pocket" or OOP or cost shar* or co-pay* or (user N1 (fee or charge) ) ) OR AB ( ( spending* OR expenditure* OR "out-of-pocket" or OOP or cost shar* or co-pay* or (user N1 (fee or charge) ) ) OR TI ( ( spending* OR expenditure* OR "out-of-pocket" or OOP or cost shar* or co-pay* or (user N1 (fee or charge) ) ) | 303598 |
| S2 | SU ( dent* OR stomato* OR teeth OR tooth OR ((dental or oral) N2 (care or service*)) OR ) OR AB ( dent* OR stomato* OR teeth OR tooth OR ((dental or oral) N2 (care or service*)) OR ) OR TI ( dent* OR stomato* OR teeth OR tooth OR ((dental or oral) N2 (care or service*)) OR ) | 37366 |
| S1 | SU ( ( (famil* or household* or person*or caregiver*) ) ) OR AB ( ( (famil* or household* or person*or caregiver*) ) ) OR TI ( ( (famil* or household* or person*or caregiver*) ) ) | 484119 |

**Cochrane**

4 Cochrane Reviews (famil* OR household* OR person* OR caregiver*) AND (((dental OR oral) NEAR/2 (care OR service*)) OR dental) AND ("out-of-pocket" OR OOP OR cost sharing OR co-payment OR (user adj (fee or charge)) OR ((financial or economic) NEAR/2 (risk or protection or hardship* or well-being or wellbeing or burden or health or toxicity or stress or pressure or difficulty or vulnerab* or satisfaction or stress or distress or challeng* or strain or worr* or problem or shock or barrier)) OR (catastroph* NEAR/3 (spend* OR expen*)) OR (bankruptcy OR debt OR impoverish* OR poverty OR afford* OR unafford*) OR (cost NEXT (barrier or high or large or excessive))) in Title Abstract Keyword - with Cochrane Library publication date from Jan to present (Word variations have been searched)

Results=7

***Grey Literature***

**WHO**

**Web page:** [*https://apps.who.int/iris/*](https://apps.who.int/iris/)

**Results: 69**

Filters : From 2000 to 2023, and English Language

Filters: MeSH term included = Health expenditures

(("family" OR person* OR "caregiv*" OR "household*") AND ("financial risk*" OR "financial protection*" OR "financial hardship*" OR "financial burden*" OR "financial toxicit*" OR "financial pressure*" OR financial difficult* OR "financial vulnerabilit*" OR "financial stress*" OR "financial distress*" OR financial challenge* OR "financial strain*" OR "financial worr*" OR "financial problem*" OR "financial shock*" OR "financial barrier*" OR "financial satisfaction*" OR "financial well-being" OR "financial wellbeing" OR "financial health" OR "economic risk*" OR "economic protection*" OR "economic hardship*" OR "economic burden*" OR "economic toxicit*" OR "economic pressure*" OR economic difficult* OR "economic vulnerabilit*" OR "economic stress*" OR "economic distress*" OR economic challenge* OR "economic strain*" OR "economic worr*" OR "economic problem*" OR "economic shock*" OR "economic barrier*" OR "economic satisfaction*" OR "economic well-being" OR "economic wellbeing" OR "economic health" OR catastroph* OR bankruptcy OR debt OR impoverish* OR "poverty" OR "cost barrier" OR "high cost" OR "large cost" OR "excessive cost") AND ("dental care" OR "dental service*") AND (spending* OR expenditure* OR "out-of-pocket" OR "OOP" OR "cost shar*" OR "co pay*" OR "user fee" OR "user charge" OR”afford*” OR “unafford*”))

**United Nations**

**Web page:** [*https://digitallibrary.un.org/*](https://digitallibrary.un.org/)

**Results: 0**

**All these words:** Dental; **Any of these words**: spending, expenditure, out-of-pocket, cost-share, co-payment, co-pay, user fee, user charge

**From:** 2005 (Jan1) -2022 (Dec 31)

**This exact phrase:**

financial hardship*= 0 results; financial burden* = 0 results; financial risk*= 0 results; financial protection*=0 results; financial stress* = 0 results; financial distress* = 0 results; financial pressure*= 0 results; financial problem*=0 results; financial difficult*=0 results; financial worr* = 0 results; financial shock* = 0 results;financial challenge* = 0 results; financial barrier* = 0 results; financial satisfaction* = 0 results; financial well-being= 0 result

financial health= 0 results; economic hardship*= 0 results; economic burden* = 0 results; economic risk*= 0 results

economic protection*=0 results; economic stress* = 0 results; economic distress* = 0 results; economic pressure*= 0 results; economic problem*=0 results; economic difficult*=0 results; economic worr* = 0 results; economic shock* = 0 results; economic challenge* = 0 results; economic barrier* = 0 results; economic satisfaction*=0 results

economic well-being= 0 results; economic health = 0 result

**World Bank Group**

**Web page:** [*https://elibrary.worldbank.org/search/advanced*](https://elibrary.worldbank.org/search/advanced)

**Results: 0**

Filters : From 2000 to 2022, and English Language

Filters: Abstract

(("family" OR person* OR "caregiv*" OR "household*") AND

("financial risk*" OR "financial protection*" OR "financial hardship*" OR "financial burden*" OR "financial toxicit*" OR "financial pressure*" OR financial difficult* OR "financial vulnerabilit*" OR "financial stress*" OR "financial distress*" OR financial challenge* OR "financial strain*" OR "financial worr*" OR "financial problem*" OR "financial shock*" OR "financial barrier*" OR "financial satisfaction*" OR "financial well-being" OR "financial wellbeing" OR "financial health" OR "economic risk*" OR "economic protection*" OR "economic hardship*" OR "economic burden*" OR "economic toxicit*" OR "economic pressure*" OR economic difficult* OR "economic vulnerabilit*" OR "economic stress*" OR "economic distress*" OR economic challenge* OR "economic strain*" OR "economic worr*" OR "economic problem*" OR "economic shock*" OR "economic barrier*" OR "economic satisfaction*" OR "economic well-being" OR "economic wellbeing" OR "economic health" OR catastroph* OR bankruptcy OR debt OR impoverish* OR "poverty" OR "cost barrier" OR "high cost" OR "large cost" OR "excessive cost") AND ("dental care" OR "dental service*") AND (spending* OR expenditure* OR "out-of-pocket" OR "OOP" OR "cost shar*" OR "co pay*" OR "user fee" OR "user charge"))

**OECD**

**Web page:** *https://www.oecd-ilibrary.org*

**Results: 10**

1 - 2 of 2 result(s)

(All Fields ‘All Fields ‘ "financial risk*" OR "financial protection*" OR “financial hardship*” OR "financial burden*" OR "financial toxicit*" OR "financial pressure*" OR financial difficult* OR "financial vulnerabilit*" OR "financial stress*" OR "financial distress*" OR financial challenge* OR "financial strain*" OR "financial worr*" OR "financial problem*" OR "financial shock*" OR "financial barrier*" OR “financial satisfaction*” OR “financial well-being” OR “financial wellbeing” OR "financial health"’) (Language ‘en’) AND (Abstract ‘dental’) AND ( ‘’) published between 1900 and 2023

Results=0

(All Fields ‘‘ "economic risk*" OR "economic protection*" OR “economic hardship*” OR " economic burden*" OR " economic toxicit*" OR " economic pressure*" OR economic difficult* OR " economic vulnerabilit*" OR " economic stress*" OR " economic distress*" OR economic challenge* OR " economic strain*" OR " economic worr*" OR " economic problem*" OR " economic shock*" OR " economic barrier*" OR “economic satisfaction*” OR “economic well-being” OR “economic wellbeing” OR " economic health" OR catastroph* OR bankruptcy OR debt OR impoverish* OR "poverty" OR "cost barrier" OR "high cost" OR "large cost" OR "excessive cost" ’ OR “afford*” OR “unafford*”’) (Language ‘en’) AND (Abstract ‘dental’) AND ( ‘’) published between 1900 and 2023

**National health agencies:**

**USA: Centers for Disease Control (CDC)**

**Web page:** [*https://www.cdc.gov/publications/index.html*](https://www.cdc.gov/publications/index.html)

**Results: 137**

**All from Journals**

**All these words:** Dental; **Any of these words**: spending, expenditure, out-of-pocket, cost-share, co-payment, co-pay, user fee, user charge

**Language:** English

**From:** 2000 (Jan1) -2023 (Dec 31)

**This exact phrase:**

financial hardship* = 5 results; financial burden* = 17 results; financial risk* = 0 results; financial protection* = 0 results; financial stress* = 6 results; financial distress* = 1 results; financial pressure* = 0 results; financial problem* = 0 results; financial difficult* = 0 results; financial worr* = 0 results; financial shock* = 0 results; financial challenge* = 2 results; financial barrier* = 2 results; financial satisfaction* = 0 results; financial well-being = 1 result; financial health = 2 results; economic hardship* = 9 results; economic burden* = 74 results; economic risk* = 0 results; economic protection* = 0 results; economic stress* = 0 results; economic distress* = 0 results; economic pressure* = 0 results; economic problem* = 0 results; economic difficult* = 0 results; economic worr* = 0 results; economic shock* = 0 results; economic challenge* = 7 results; economic barrier* = 4 result; economic satisfaction* = 0 results; economic well-being = 6 results; economic health = 4 result.

Duplicates found: 8

**Canada: Health Canada**

**Web page:** <https://www.canada.ca/en/sr/srb/sra.html>

**Results: 25**

**All these words:** Dental; **Any of these words**: spending, expenditure, out-of-pocket, cost-share, co-payment, co-pay, user fee, user charge

**Language: English**

**Site or domain:** publications

**This exact phrase:**

financial hardship* = 10 results; financial burden* = 9 results; financial risk* = 0 results; financial protection* = 0 results; financial stress* = 0 results; financial distress* = 0 results; financial pressure* = 2 results; financial problem* = 0 results; financial difficult* = 0 results; financial worr* = 0 results; financial shock* = 0 results; financial challenge* = 0 results; financial barrier* = 0 results; financial satisfaction* = 0 results; financial well-being = 0 result; financial health = 0 results; economic hardship* = 0 results; economic burden* = 0 results; economic risk* = 0 results; economic protection* = 0 results; economic stress* = 0 results; economic distress* = 0 results; economic pressure* = 0 results; economic problem* = 0 results; economic difficult* = 0 results; economic worr* = 0 results; economic shock* = 0 results; economic challenge* = 0 results; economic barrier* = 4 results; economic satisfaction* = 0 results; economic well-being = 0 results; economic health = 0 result.

**Australia: Department of Health and Aged care**

**Web page:** [*https://www.health.gov.au/resources/publications*](https://www.health.gov.au/resources/publications)

**Results: 0**

Search: Dental AND

financial hardship* = 0 results; financial burden* = 0 results; financial risk* = 0 results; financial protection* = 0 results; financial stress* = 0 results; financial distress* = 0 results; financial pressure* = 0 results; financial problem* = 0 results; financial difficult* = 0 results; financial worr* = 0 results; financial shock* = 0 results; financial challenge* = 0 results; financial barrier* = 0 results; financial satisfaction* = 0 results; financial well-being = 0 result; financial health = 0 results; economic hardship* = 0 results; economic burden* = 0 results; economic risk* = 0 results; economic protection* = 0 results; economic stress* = 0 results; economic distress* = 0 results; economic pressure* = 0 results; economic problem* = 0 results; economic difficult* = 0 results; economic worr* = 0 results; economic shock* = 0 results; economic challenge* = 0 results; economic barrier* = 0 results; economic satisfaction* = 0 results; economic well-being = 0 results; economic health = 0 result.

**New Zealand: Ministry of health**

**Web page:** [*https://www.health.govt.nz/publications/*](https://www.health.govt.nz/publications/)

**Results: 0**

Search: Dental AND

financial hardship* = 0 results; financial burden* = 0 results; financial risk* = 0 results; financial protection* = 0 results; financial stress* = 0 results; financial distress* = 0 results; financial pressure* = 0 results; financial problem* = 0 results; financial difficult* = 0 results; financial worr* = 0 results; financial shock* = 0 results; financial challenge* = 0 results; financial barrier* = 0 results; financial satisfaction* = 0 results; financial well-being = 0 result; financial health = 0 results; economic hardship* = 0 results; economic burden* = 0 results; economic risk* = 0 results; economic protection* = 0 results; economic stress* = 0 results; economic distress* = 0 results; economic pressure* = 0 results; economic problem* = 0 results; economic difficult* = 0 results; economic worr* = 0 results; economic shock* = 0 results; economic challenge* = 0 results; economic barrier* = 0 results; economic satisfaction* = 0 results; economic well-being = 0 results; economic health = 0 result.

**UK: England (Department of health and social care)**

**Results**: 2

**Web page:** <https://www.gov.uk/search/policy-papers-and-consultations?parent=department-of-health-and-social-care>

**About**: Health and Social care AND research and innovation in health and social care

**From**: Department of Health and Social Care

**Updated between**: January 1, 2000 to December 31, 2023

**Search**: Dental AND

financial hardship* = 0 results; financial burden* = 1 results; financial risk* = 0 results; financial protection* = 0 results; financial stress* = 0 results; financial distress* = 0 results; financial pressure* = 0 results; financial problem* = 0 results; financial difficult* = 0 results; financial worr* = 0 results; financial shock* = 0 results; financial challenge* = 0 results; financial barrier* = 1 results; financial satisfaction* = 0 results; financial well-being = 0 result; financial health = 0 results; economic hardship* = 0 results; economic burden* = 0 results; economic risk* = 0 results; economic protection* = 0 results; economic stress* = 0 results; economic distress* = 0 results; economic pressure* = 0 results; economic problem* = 0 results; economic difficult* = 0 results; economic worr* = 0 results; economic shock* = 0 results; economic challenge* = 0 results; economic barrier* = 0 results; economic satisfaction* = 0 results; economic well-being = 0 results; economic health = 0 result.

**UK: Scotland (Public Health Scotland)**

**Web page:** [*https://publichealthscotland.scot/publications/*](https://publichealthscotland.scot/publications/)

**Results: 0**

Search: Dental AND

financial hardship* = 0 results; financial burden* = 0 results; financial risk* = 0 results; financial protection* = 0 results; financial stress* = 0 results; financial distress* = 0 results; financial pressure* = 0 results; financial problem* = 0 results; financial difficult* = 0 results; financial worr* = 0 results; financial shock* = 0 results; financial challenge* = 0 results; financial barrier* = 0 results; financial satisfaction* = 0 results; financial well-being = 0 result; financial health = 0 results; economic hardship* = 0 results; economic burden* = 0 results; economic risk* = 0 results; economic protection* = 0 results; economic stress* = 0 results; economic distress* = 0 results; economic pressure* = 0 results; economic problem* = 0 results; economic difficult* = 0 results; economic worr* = 0 results; economic shock* = 0 results; economic challenge* = 0 results; economic barrier* = 0 results; economic satisfaction* = 0 results; economic well-being = 0 results; economic health = 0 result.

**UK: Wales (Public Health Wales)**

**Web page:** [*https://phw.nhs.wales/publications/*](https://phw.nhs.wales/publications/)

**Results: 0**

financial hardship* = 0 results; financial burden* = 0 results; financial risk* = 0 results; financial protection* = 0 results; financial stress* = 0 results; financial distress* = 0 results; financial pressure* = 0 results; financial problem* = 0 results; financial difficult* = 0 results; financial worr* = 0 results; financial shock* = 0 results; financial challenge* = 0 results; financial barrier* = 0 results; financial satisfaction* = 0 results; financial well-being = 0 result; financial health = 0 results; economic hardship* = 0 results; economic burden* = 0 results; economic risk* = 0 results; economic protection* = 0 results; economic stress* = 0 results; economic distress* = 0 results; economic pressure* = 0 results; economic problem* = 0 results; economic difficult* = 0 results; economic worr* = 0 results; economic shock* = 0 results; economic challenge* = 0 results; economic barrier* = 0 results; economic satisfaction* = 0 results; economic well-being = 0 results; economic health = 0 result.

**UK: Northern Ireland (Public Health Northern Ireland)**

**Web page:** [*https://www.publichealth.hscni.net*](https://www.publichealth.hscni.net)

**Results: 0**

financial hardship* = 0 results; financial burden* = 0 results; financial risk* = 0 results; financial protection* = 0 results; financial stress* = 0 results; financial distress* = 0 results; financial pressure* = 0 results; financial problem* = 0 results; financial difficult* = 0 results; financial worr* = 0 results; financial shock* = 0 results; financial challenge* = 0 results; financial barrier* = 0 results; financial satisfaction* = 0 results; financial well-being = 0 result; financial health = 0 results; economic hardship* = 0 results; economic burden* = 0 results; economic risk* = 0 results; economic protection* = 0 results; economic stress* = 0 results; economic distress* = 0 results; economic pressure* = 0 results; economic problem* = 0 results; economic difficult* = 0 results; economic worr* = 0 results; economic shock* = 0 results; economic challenge* = 0 results; economic barrier* = 0 results; economic satisfaction* = 0 results; economic well-being = 0 results; economic health = 0 result.

**Ireland (Department of Health)**

**Web page:** [*https://www.gov.ie/en/publications/*](https://www.gov.ie/en/publications/)

**Results from:** Department of Finance × Department of Health × Department of Social Protection × Health Research Board (HRB) × Health Service Executive × Healthy Ireland

**Results: 41**

**Filter: Publications, Department of Health**

financial hardship* = 3 results; financial burden* = 0 results; financial risk* = 6 results; financial protection* = 6 results; financial stress* = 0 results; financial distress* = 1 result; financial pressure* = 1 result; financial problem* = 0 results; financial difficult* = 0 results; financial worr* = 0 results; financial shock* = 0 results; financial challenge* = 1 result; financial barrier* = 1 result; financial satisfaction* = 0 results; financial well-being = 5 results; financial health = 1 results; economic hardship* = 0 results; economic burden* = 2 result; economic risk* = 0 results; economic protection* = 0 results; economic stress* = 0 results; economic distress* = 0 results; economic pressure* = 0 results; economic problem* = 0 results; economic difficult* = 0 results; economic worr* = 0 results; economic shock* = 1 result; economic challenge* = 4 results; economic barrier* = 0 results; economic satisfaction* = 0 results; economic well-being = 7 results; economic health = 3 results.

**ProQuest Dissertations and Thesis Global**

**Results: 228**

("family" OR "persons" OR "caregiv*" OR "famil*" OR "person*" OR "household*" OR "adult*") AND noft(("financial risk*" OR "financial protection*" OR "financial hardship*" OR "financial burden*" OR "financial toxi*" OR "financial stress*" OR "financial pressure*" OR "financial difficult*" OR "financial vulner*" OR "financial distress*" OR "financial challenge*" OR "financial strain*" OR "financial worr*" OR "financial problem*" OR "financial shock*" OR "financial barrier*" OR "financial satisfaction" OR "financial well-being" OR "financial wellbeing" OR "financial health" OR "economic risk*" OR "economic protection*" OR "economic hardship*" OR "economic toxic*" OR "economic stress*" OR "economic pressure*" OR "economic difficult*" OR "economic vulner*" OR "economic distress*" OR "economic challenge*" OR "economic strain*" OR "economic worr*" OR "economic problem*" OR "economic shock*" OR "economic barrier*" OR "economic satisfaction*" OR "economic well-being" OR "economic health" OR "catastroph*" OR "bankruptcy" OR "debt*" OR "impoverish*" OR "poverty" OR "cost barrier*" OR "high cost*" OR "large cost*" OR "excessive cost*")) AND noft(("dental care" OR "dental health services" OR "dental care" OR "oral care" OR "dental service*" OR "oral service*" OR "stomato*" OR "dent*" OR "teeth" OR "tooth")) AND ("health expenditures" OR "expenditure*" OR "spending*" OR "out-of-pocket" OR "out-of-pocket" OR "OOP" OR "cost shar*" OR "co pay*" OR "user fee" OR "user charge" OR "afford*" OR "unafford*")

Filters: English, time from 2000-2023

**Appendix Table 1:** List of publications included

| **First author, publication year** | **Population** | **Study period** | **Publication type** |
| --- | --- | --- | --- |
| Peck, 2006 | Households | 2004 | Published study |
| Pérez-Nuñez, 2007 | Households | 2000 to 2004 | Published study |
| WHO, 2009 | Households | 1995 to 2007 | Grey literature |
| Muirhead, 2009 | Adults | 2007 | Published study |
| Snow, 2010 | Households | 2006/07 | Published study |
| WHO, 2010 | Households | 1994/95 to 2005/06 | Grey literature |
| Kim, 2011 | Households | 2006 | Published study |
| Locker, 2011 | Adults | 2008 | Published study |
| Kavosi, 2012 | Households | 2003 to 2008 | Published study |
| MacEntee, 2012 | Seniors | 2010 | Published study |
| Chrisopoulos, 2013 | Adults | 2010 | Grey literature |
| Rahman, 2013 | Households | 2011 | Published study |
| ARCPOH, 2014 | Children | 2010 | Published study |
| Himmelstein, 2014 | Adults | 2008 and 2010 | Published study |
| Kavosi, 2014 | Households | 2012 | Published study |
| Masood, 2015 | Households | 2002 to 2004 | Published study |
| Sun, 2016 | Households | 2005 | Published study |
| Willink, 2016 | Adults | 2012 | Published study |
| Bernabé, 2017 | Households | 2002 to 2004 | Published study |
| Moradi, 2017 | Households^a^ | 2015 | Published study |
| Cooke O’Dowd, 2018 | Households | 2012 to 2015 | Grey literature |
| Czypionka, 2018* | Households | 2004/05 to 2014/15 | Grey literature |
| Goroshko, 2018* | Households | 2010 to 2015 | Grey literature |
| Jakab, 2018* | Households | 2000 to 2014 | Grey literature |
| Murauskienė, 2018* | Households | 2005 to 2012 | Grey literature |
| Proaño Falconí, 2018 | Households | 2016 | Published study |
| Siegel, 2018* | Households | 2008 to 2016 | Grey literature |
| Thomson, 2018* | Households | 2012, 2013, 2015 | Grey literature |
| Taube, 2018* | Households | 2008 to 2013 | Grey literature |
| Vončina, 2018* | Households | 2010 to 2014 | Grey literature |
| Võrk, 2018* | Households | 2000 to 2015 | Grey literature |
| AlBaty, 2019 | Adults | 2018 | Published study |
| Barfar, 2019 | Households^c^ | 2017/18 | Published study |
| Edmonds, 2019 | Households | 2010 to 2015 | Published study |
| Ghorbanian 2019 | Households | 1995 to 2015 | Published study |
| Glenngård, 2019* | Households | 2006 to 2012 | Grey literature |
| Nyamuryekung'e, 2019 | Adults | 2016 | Published study |
| Mehdizadeh, 2019 | Households^b^ | 2016 | Published study |
| Peltz, 2019 | Households | 2016 | Published study |
| Quintal, 2019* | Households | 2015/16 | Grey literature |
| Thomson, 2019* | Households | 2011 to 2016 | Grey literature |
| Garam, 2020* | Households | 2008 to 2016 | Grey literature |
| Johnston, 2020* | Households | 2009/10 to 2015/16 | Grey literature |
| Khammarnia, 2020 | Households | 2017 | Published study |
| Nemati, 2020 | Households | 2017 | Published study |
| Nobelika, 2020 | Sanitary workers | 2018 | Published study |
| Tambor, 2020* | Households | 2005 to 2014 | Grey literature |
| Tomini, 2020* | Households | 2008/09 to 2015 | Grey literature |
| Ahmadi, 2021 | Households | 2011 and 2020 | Published study |
| Hsu, 2021 | Households | 2005 to 2017 | Published study |
| Kontemeniotis, 2021* | Households | 2003 to 2015 | Grey literature |
| Prasad, 2021 | Households | 2014 and 2017/18 | Published study |
| Uguru, 2021 | Households | 2018 | Published study |
| Urbanos-Garrido, 2021* | Households | 2006 to 2019 | Grey literature |
| Tervola, 2021* | Households | 2006 to 2016 | Grey literature |
| Woldemichael, 2021 | Households | 2018 | Published study |
| Aeenparast, 2022 | Adults | 2019 | Published study |
| Lee, 2022 | Households | 2016 and 2017 | Published study |
| Liu, 2022 | Veterans | 2010 to 2017 | Published study |
| López-López, 2022 | Households | 2008 to 2015 | Published study |
| Scîntee, 2022* | Households | 2010 to 2015 | Grey literature |
| Vojvodic, 2022 | Seniors | 2017 | Published study |
| Boukaert, 2023 | Households | 2012 to 2020 | Grey literature |
| Shokri, 2023 | Households | 2021 | Published study |
| López-López, 2023 | Households | 2008 to 2015 | Published study |

WHO: World Health Organization; ARCPOH: Australian Research Centre for Population Oral Health.*WHO Regional Office for Europe reports. ^a^ Members with multiple sclerosis, dialysis, or kidney transplant among family members; ^b^ Health staff households; ^c^ Members with severe mental disorder patients.

**Appendix Table 2:** Study summary characteristics and references

| **Measures/ characteristics** | | **References** |
| --- | --- | --- |
| **1. Catastrophic spending** | |  |
| **1.1 Catastrophic health expenditure** *(n=43)* | |  |
| - *Country income group^a^* | - *High-income (n=24)* | (1–24) |
|  | - *Upper-middle income (n=3)* | (25–27) |
|  | - *Lower-middle income (n=14)* | (28–41) |
|  | - *High & upper-and-lower-middle income (n=1)* | (42) |
|  | - *Upper-and-lower middle income and low-income (n=1)* | (43) |
|  |  |  |
| - *Data collection* | - *Primary data collection* *(n=9)* | (24,28–34,36) |
|  | - *Secondary data collection (n=34)* | (1–23,25–27,35,37–43) |
|  |  |  |
| - *Study design* | - *Systematic review* *(n=1)* | (37) |
|  | - *Cross-sectional (n=47)* | (1–36,38–43) |
|  |  |  |
| - *Instrument^b^* | - *National household spending survey (n=28)* | (1–3,5–21,23,25–27,35,38–42) |
|  | - *Health care spending survey* *(n=2)* | (4,22) |
|  | - *Household health survey* *(n=9)* | (24,28–34,36,43) |
| **1.2 Catastrophic dental health expenditure** *(n=10)* | |  |
| - *Country income group^b^* | - *High-income (n=4)* | (44–47) |
|  | - *Upper-middle income (n=2)* | (48,49) |
|  | - *Lower-middle income (n=3)* | (50–52) |
|  | - *Upper-and-lower middle income and low-income (n=1)* | (53) |
|  |  |  |
| - *Data collection* | - *Primary data collection (n=3)* | (46,50,52) |
|  | - *Secondary data collection (n=7)* | (44,45,47–49,51,53) |
|  |  |  |
| - *Study design* | - *Cross-sectional (n=10)* | (44–53) |
|  |  |  |
| - *Instruments* | - *Author-created questionnaire (n=2)* | (50,52) |
|  | - *National household spending survey (n=3)* | (44,47,48) |
|  | - *National oral health survey (n=2)* | (49,51) |
|  | - *Household health survey* *(n=1)* | (53) |
|  | - *Dental charts (n=1)* | (45) |
|  | - *Unclear (n=1)* | (46) |
| **2. Impoverishment** *(n=1)* | |  |
| - *Country income group^b^* | - *Upper-and-lower middle income and low-income (n=1)* | (43) |
|  |  |  |
| - *Data collection* | - *Secondary data collection* *(n=1)* | (43) |
|  |  |  |
| - *Study design* | - *Cross-sectional* *(n=1)* | (43) |
|  |  |  |
| - *Instruments* | - *Household health survey* *(n=1)* | (43) |

| **3. Self-reported financial burden from OHC** *(n=6)* | |  |
| --- | --- | --- |
| - *Country income group^b^* | - *High-income (n=3)* | (54–56) |
|  | - *Lower-middle income (n=2)* | (52,57) |
|  | - *High & upper-middle income (n=1)* | (58) |
|  |  |  |
| - *Data collection* | - *Primary data collection (n= 4)* | (52,54,56,57) |
|  | - *Secondary data collection (n= 2)* | (55,58) |
|  |  |  |
| - *Study design* | - *Cross-sectional (n=6)* | (52,54–58) |
|  |  |  |
| - *Instruments* | - *Author-created questionnaire (n=2)* | (52,54) |
|  | - *Household survey (n=1)* | (58) |
|  | - *National oral health survey (n=2)* | (55,56) |
|  | - *Unclear (n=1)* | (57) |
| **4. Distress financing** *(n=1)* | |  |
| - *Country income group^b^* | - *Lower-middle income (n=1)* | (59) |
|  |  |  |
| - *Data collection* | - *Primary data collection* *(n=1)* | (59) |
|  |  |  |
| - *Study design* | - *Cross-sectional* *(n=1)* | (59) |
|  |  |  |
| - *Instrument* | - *Author-created questionnaire (n=1)* | (59) |
| **5. Bankruptcy** *(n=1)* | |  |
| - *Country income group^b^* | - *High-income (n=1)* | (60) |
|  |  |  |
| - *Data collection* | - *Primary data collection* *(n=1)* | (60) |
|  |  |  |
| - *Study design* | - *Cross-sectional* *(n=1)* | (60) |
|  |  |  |
| - *Instrument* | - *Author-created questionnaire (n=1)* | (60) |
| **6. Self-reported food insecurity** *(n=3)* | |  |
| - *Country income group^b^* | - *High-income (n=3)* | (61–63) |
|  |  |  |
| - *Data collection* | - *Primary data collection (n=1)* | (61) |
|  | - *Secondary data collection (n=2)* | (62,63) |
|  |  |  |
| - *Study design* | - *Cross-sectional (n=3)* | (61–63) |
|  |  |  |
| - *Instruments* | - *National health care spending survey and food insecurity questionnaire* | (62,63) |
|  | - *Author-created questionnaire and food insecurity questionnaire* | (61) |
| **7. Financial hardship experience from paying out-of-pocket for OHC** *(n=2)* | | |
| - *Country income group^b^* | - *High-income (n=2)* | (64,65) |
|  |  |  |
| - *Data collection* | - *Primary data collection (n=2)* | (64,65) |
|  |  |  |
| - *Study design* | - *Qualitative design (n=2)* | (64,65) |
|  |  |  |
| - *Instruments* | - *Narrative inquiry (n=1)* | (64) |
|  | - *Focus group (n=1)* | (65) |

*Two studies used more than 1 financial hardship measure, so the total number of studies shown (67) is greater than the number of studies included in our review (65). OOP, out-of-pocket; OHC, oral health care. Three studies used two different financial hardship measures. ^a^From the 2023 World Bank Group country income group list. ^b^Excludes systematic review*

Appendix Table 3: Comparison between the different financial hardship in OHC concepts employed in the literature

|  | **Catastrophic spending** | | **Impoverishment (IMPOV)** | **Financial burden** | **Negative coping strategies** | **Bankruptcy** | **Food insecurity** |
| --- | --- | --- | --- | --- | --- | --- | --- |
|  | **Catastrophic health expenditure (CHE)** | **Catastrophic dental health expenditure (CDHE)** |  |  |  |  |  |
| **CDHE** | *Similarity*:  Both identify people employing a large fraction of their income on health care OOP spending (including OHC).  *Difference*:  CDHE is specific for only OHC services while CHE may include different health care services and OHC is not necessarily included. |  |  |  |  |  |  |
| **Impoverishment (IMPOV)** | *Similarity*:  Both identify people spending a large fraction of their household’s income on health care (including OHC).  *Difference*:  IMPOV quantifies if the household is being pushed over the poverty line due to OHC OOP spending. CHE does not imply IMPOV, and IMPOV may not necessarily imply CHE. | *Similarity*:  Both identify a household spending a large amount on OHC OOP relative to their income  *Difference*  CDHE only occurs with OHC OOP spending while IMPOV includes other OHC OOP spending (not necessarily OHC). IMPOV is related to poverty caused by health care OOP spending. |  |  |  |  |  |
| **Financial burden** | *Similarity*:  Both identify a financial hardship situation from paying OOP for OHC.  *Difference*:  Financial burden is specific to OHC. CHE can include more than one health care service and does not necessarily include OHC. Further, CHE can quantify the extent of financial hardship. | *Similarity*:  Both are specific to OHC OOP spending.  *Difference*:  CDHE quantifies how much is the household’s finances being compromised for paying OHC OOP. Financial burden provides how people self-assess the financial hardship from OHC OOP spending. | *Similarity*:  Both may identify a financial hardship situation from paying OOP for OHC.  *Difference*:  IMPOV can include other health care OOP spending and not necessarily OHC. Financial burden is specific to OHC OOP spending. |  |  |  |  |
| **Negative coping strategies** | *Similarity*:  Both concepts may include other health care services and are not specific to only OHC OOP spending.  *Difference*:  Negative coping strategies may include other forms of financial hardship (e.g., distress financing: extra working hours, loans or removing children from school) that are not captured in CHE measurements. CHE may occur among households with higher income/consumption than those employing negative coping strategies. | *Similarity*:  Both concepts may capture people having to pay large amounts for OHC OOP.  *Difference*:  Negative coping strategies include other forms of financial hardship (e.g., distress financing: extra working hours, loans or removing children from school) that are not captured in CDHE and may include other health care OOP spending. CDHE may occur among households with higher income/consumption than those experiencing distress financing. | *Similarity*:  Both concepts may include other health care services and are not specific to only OHC OOP spending. Also, those recurring to some negative coping strategies (e.g., reducing food spending) may be at risk of IMPOV.  *Difference*:  Negative coping strategies can be measured as distress financing (e.g., extra working hours, loans or removing children from school) that are not captured in IMPOV measurements. On the other hand, IMPOV quantifies the impact of OHC OOP spending. | *Similarity*:  Both concepts may capture people having to pay large amounts for OHC OOP.  *Difference*:  Negative coping strategies include other forms of financial hardship (e.g., distress financing: extra working hours, loans or removing children from school) that are not captured in financial burden and may include other health care OOP spending. Financial burden may occur in the absence of distress financing. |  |  |  |
| **Bankruptcy** | *Similarity*:  Both may identify people having financial struggles and having to pay a large amount of their household’s income on health care OOP (including OHC).  *Difference*:  Bankruptcy is a broader concept and is not bound to health care OOP spending.  CHE is more specific in quantifying the impact of health care OOP but doesn’t identify people having trouble with their debts specifically. | *Similarity*:  Both may identify a household with debts they can’t keep up with and consuming a large amount of their household’s financial resources on OHC OOP spending.  *Difference*:  Bankruptcy is a broader concept and is not bound to health care OOP spending.  CDHE is more specific in quantifying the impact OHC OOP has on the household’s financial resources, and does not occur only among those with debts. | *Similarity*:  Both may identify people having financial struggles and having to pay a large amount of their household’s income on health care OOP (including OHC).  *Difference*:  Bankruptcy is a broader concept and is not bound to health care OOP spending.  IMPOV may occur in cases of bankruptcy or not. | *Similarity*:  Both may identify a household with debts they can’t keep up with and consuming a large amount of their household’s financial resources on OHC OOP spending.  *Difference*:  Bankruptcy is a broader concept and is not bound to health care OOP spending.  Financial burden is specific to oral health OOP spending. | *Similarity*:  Both may identify people having financial struggles and having to pay a large amount of their household’s income on health care OOP (including OHC). Both include the selling of assets.  *Difference*:  Bankruptcy is a broader concept and is not bound to health care OOP spending.  Negative coping strategies include other forms of financial hardship (e.g., extra working hours, loans or removing children from school) that are not captured in bankruptcy. |  |  |
| **Food insecurity** | *Similarity*:  Both may identify people having financial struggles and having to pay a large amount of their household’s income on health care OOP (including OHC).  *Difference*:  Food insecurity is a broader concept that not necessarily is affected by health care OOP spending. CHE can quantify the effect of health care (including OHC) on the household’s finances. | *Similarity*:  Both may identify people having financial struggles and having to pay a large amount of their household’s income on OHC OOP.  *Difference*:  Food insecurity is a broader concept that not necessarily is affected by OHC OOP spending. CDHE can quantify the effect of OHC on the household’s finances. | *Similarity*:  Both may capture people having trouble paying for their food needs and having to pay for health care OOP (including OHC).  *Difference*:  Food insecurity is a broader concept that not necessarily is affected by health care OOP spending. IMPOV quantifies the impact of paying OOP for health care. IMPOV can be reflective of national/international poverty lines, which include other goods. | *Similarity*:  Both may identify people having financial struggles and having to pay a large amount of their household’s income on OHC OOP.  *Difference*:  Food insecurity is a broader concept that not necessarily is affected by OHC OOP spending. Financial burden only occurs among those spending OOP for OHC services. | *Similarity*:  Both may identify people having financial struggles and having to pay a large amount of their household’s income on health care OOP (including OHC).  *Difference*:  Food insecurity is a broader concept that not necessarily is affected by health care OOP spending. Negative coping strategies include other forms of financial hardship (e.g., distress financing: extra working hours, loans or removing children from school) that are not captured in food insecurity. | *Similarity*:  Both are broader concepts of financial hardship.  *Difference*:  Food insecurity is not contingent on people keeping up (or having) debts. People who are experiencing bankruptcy may not necessarily be experiencing food insecurity. |  |
| **Personal experience** | *Similarity*:  Both may identify people having to spend a large amount on OHC relative to their income.  *Difference*:  Personal experience of financial hardship from OHC is specific to OHC OOP spending. CHE can include other health care OOP spending and not necessarily does it include OHC. CHE can quantify the impact of OHC OOP on the household’s income. | *Similarity*:  Both are specific to spending OOP on OHC services.  *Difference*:  Personal experience can provide a more nuanced understanding of why the person is experiencing financial hardship from OHC OOP spending. CDHE can quantify the impact of OHC OOP on the household’s income. | *Similarity*:  Both may capture people experiencing financial struggles and having to pay for OHC OOP.  *Difference*:  Personal experience can provide a more nuanced understanding of why the person is experiencing financial hardship from OHC OOP spending.  IMPOV quantifies if the household is being pushed over the poverty line due to health care OOP spending (including OHC). | *Similarity*:  Both are specific to spending OOP on OHC services.  *Difference*:  Personal experience can provide a more nuanced understanding of why the person is experiencing financial hardship from OHC OOP spending. Financial burden can differentiate between a “large” “moderate” or “mild” experience of financial hardship. | *Similarity*:  Both may capture people experiencing financial struggles and having to pay for OHC OOP.  *Difference*:  Personal experience of financial hardship from OHC is specific to OHC OOP spending. Negative coping strategies may include other health care services as is not limited to OHC OOP spending. | *Similarity*:  Both may identify people having to spend a large amount on OHC relative to their income.  *Difference*:  Personal experience of financial hardship from OHC is specific to OHC OOP spending. Bankruptcy is a broader concept of financial hardship that does not imply OOP costs for health care or OHC. | *Similarity*:  Both may identify people having to spend a large amount on OHC relative to their income.  *Difference*:  Personal experience of financial hardship from OHC is specific to OHC OOP spending. Food insecurity is a broader concept of financial hardship that does not imply OOP costs for health or OHC. |

OOP, out-of-pocket; OHC, oral health care

**Appendix Table 4:** Study definition of oral health care (OHC) out-of-pocket (OOP), recall period for use of OHC, and OHC services consumed

| **Measures/studies** | **Definition of OHC OOP** | **Instrument** | **Recall period** | **OHC services** |
| --- | --- | --- | --- | --- |
| **1.1 Catastrophic health expenditure** | |  |  | |
| WHO 2009 | Direct payments | National household spending survey | Unclear | OHC |
| WHO 2010 | Excludes reimbursement | National household spending survey | 6 months and 12 months | OHC |
| Kim 2011 | Direct payments | National household spending survey | 1 month | OHC |
| Kavosi 2012 | Cash or kind | Household health survey (*World Health Survey*) | 1 month | OHC |
| Kavosi 2014 | Direct payments | Household health survey (*World Health Survey*) | 1 month | OHC |
| Willink 2016 | Direct payments | Healthcare spending survey (*Medicare Surv*ey) | 12 months | OHC |
| Bernabé 2017 | Cash or kind. No reimbursement. | Household health survey (*World Health Survey*) | 1 month | OHC |
| Moradi 2017 | Direct payments | Household health survey (*World Health Survey*) | 1 month | OHC |
| Cooke O’Dowd 2018* | Includes formal co-payment, formal payments, and informal payments. Excludes pre-payment and reimbursement of the household by a third party. | National household spending survey | Unclear | OHC |
| Czypionka 2018* | Includes formal co-payment, formal payments, and informal payments. Excludes pre-payment and reimbursement of the household by a third party. | National household spending survey | Unclear | OHC |
| Goroshko 2018* | Includes formal co-payment, formal payments, and informal payments. Excludes pre-payment and reimbursement of the household by a third party. | National household spending survey | Unclear | OHC |
| Jakab 2018* | Includes formal co-payment, formal payments, and informal payments. Excludes pre-payment and reimbursement of the household by a third party. | National household spending survey | Unclear | OHC |
| Murauskienė 2018* | Includes formal co-payment, formal payments, and informal payments. Excludes pre-payment and reimbursement of the household by a third party. | National household spending survey | Unclear | OHC |
| Proaño Falconí 2018 | Direct payments | National household spending survey | 3 months | OHC |
| Siegel 2018* | Includes formal co-payment, formal payments, and informal payments. Excludes pre-payment and reimbursement of the household by a third party. | National household spending survey | Unclear | OHC |
| Taube 2018* | Includes formal co-payment, formal payments, and informal payments. Excludes pre-payment and reimbursement of the household by a third party. | National household spending survey | Unclear | OHC |
| Thomson 2018* | Includes formal co-payment, formal payments, and informal payments. Excludes pre-payment and reimbursement of the household by a third party. | National household spending survey | Unclear | OHC |
| Vončina 2018* | Includes formal co-payment, formal payments, and informal payments. Excludes pre-payment and reimbursement of the household by a third party. | National household spending survey | Unclear | OHC |
| Võrk 2018* | Includes formal co-payment, formal payments, and informal payments. Excludes pre-payment and reimbursement of the household by a third party. | National household spending survey | Unclear | OHC |
| Barfar 2019 | Direct payments | Household health survey (*World Health Survey*) | 1 month | OHC |
| Edmonds 2019 | Excludes insurance payments | National household spending survey | 12 months | OHC |
| Ghorbanian 2019 | Includes cost-sharing payments | NA | NA | OHC |
| Glenngård 2019* | Includes formal co-payment, formal payments, and informal payments. Excludes pre-payment and reimbursement of the household by a third party. | National household spending survey | Unclear | OHC |
| Mehdizadeh 2019 | Direct payments | World Health Survey | 1 month | OHC |
| Quintal 2019 | Direct payments | National household spending survey | 12 months | OHC |
| Thomson 2019* | Includes formal co-payment, formal payments, and informal payments. Excludes pre-payment and reimbursement of the household by a third party. | National household spending survey | Unclear | OHC |
| Garam 2020* | Includes formal co-payment, formal payments, and informal payments. Excludes pre-payment and reimbursement of the household by a third party. | National household spending survey | Unclear | OHC |
| Johnston 2020* | Includes formal co-payment, formal payments, and informal payments. Excludes pre-payment and reimbursement of the household by a third party. | National household spending survey | Unclear | OHC |
| Khammarnia 2020 | Direct payments | Household health survey (*World Health Survey*) | 1 month | OHC |
| Nemati 2020 | Direct payments | Household health survey (*World Health Survey*) | 1 month | OHC |
| Tambor 2020* | Includes formal co-payment, formal payments, and informal payments. Excludes pre-payment and reimbursement of the household by a third party. | National household spending survey | Unclear | OHC |
| Tomini 2020* | Includes formal co-payment, formal payments, and informal payments. Excludes pre-payment and reimbursement of the household by a third party. | National household spending survey | Unclear | OHC |
| Ahmadi 2021 | Direct payments | Household health survey (*World Health Survey*) | 1 month | OHC |
| Hsu 2021 | Direct payments | National household spending survey | 12 months | OHC |
| Kontemeniotis 2021* | Includes formal co-payment, formal payments, and informal payments. Excludes pre-payment and reimbursement of the household by a third party. | National household spending survey | Unclear | OHC |
| Tervola 2021* | Includes formal co-payment, formal payments, and informal payments. Excludes pre-payment and reimbursement of the household by a third party. | National household spending survey | Unclear | OHC |
| Urbanos-Garrido 2021* | Includes formal co-payment, formal payments, and informal payments. Excludes pre-payment and reimbursement of the household by a third party. | National household spending survey | Unclear | OHC |
| Woldemichael 2021 | Direct payments | National household spending survey | 1 month | OHC |
| Aeenparast 2022 | Direct payments | National household spending survey | 12 months | OHC |
| Liu 2022 | Direct payments | Healthcare spending survey (*MEPS*) | 12 months | OHC |
| Scîntee 2022* | Includes formal co-payment, formal payments, and informal payments. Excludes pre-payment and reimbursement of the household by a third party. | National household spending survey | Unclear | OHC |
| Boukaert, 2023 | Includes formal co-payment, formal payments, and informal payments. Excludes pre-payment and reimbursement of the household by a third party. | National household spending survey | Unclear | OHC |
| Shokri, 2023 | Direct payments | Household health survey (*World Health Survey*) | 1 month | OHC |
| **1.2 Catastrophic dental health expenditure** | |  |  | |
| Pérez-Nuñez 2007 | Cash or kind | National household spending survey | 3 months | OHC |
| Snow 2010 | Direct payments | Dental charts | 12 months | Preventive, diagnostic, restorative, periodontal, orthodontics, endodontic, denture services, minor surgery |
| Masood 2015 | Cash or kind. No reimbursement. | National health survey (*World Health Surve*y) | 1 month | OHC |
| Sun 2016 | Direct payments | National household spending survey | 12 months | Medicines, OHC |
| Nyamuryekung'e 2019 | Direct payments | Author-created questionnaire | 4-5 weeks | Diagnostic, periodontal, extraction, radiographic services, other dental services |
| AlBaty 2019 | Direct payments | Unclear | 12 months | Medicines, preventive, restorative, periodontal, aesthetic, endodontic, extraction, denture services |
| Nobelika 2020 | Direct payments | Author-created questionnaire | 12 months | Medicines, OHC |
| Prasad 2021 | Direct payments | National household spending survey | 15 days | Medicines, inpatient, diagnostic, other services |
| López-López 2022 | Direct payments | National household spending survey | 12 months | OHC |
| López-López 2023 | Direct payments | National household spending survey | 12 months | OHC |
| **2. Impoverishment** |  |  |  |  |
| Bernabe 2017 | Cash or kind. No reimbursement. | National household spending survey | 1 month | OHC |
| **3. Self-reported financial burden from OHC** | |  |  | |
| Locker 2011 | Direct payments | Author-created questionnaire | 3 years | OHC |
| Chrisopoulos 2013 | Direct payments | National oral health survey | 12 months | OHC |
| ARCPOH 2014 | Direct payments | National oral health survey | 12 months | Preventive, diagnostic, restorative, orthodontics, extraction, radiographic services |
| Nobelika 2020 | Direct payments | Author-created questionnaire | 12 months | Medicines, OHC |
| Uguru 2021 | Direct payments | Unclear | 6 months | Restorative services |
| Vojvodic 2022 | No insurance payments | Household survey (*European Union Survey on Statistics on Income and Living Conditions*) | 12 months | Preventive, diagnostic, restorative, orthodontics |
| **4. Distress financing** |  |  |  |  |
| Rahman 2013 | Direct payments | Author-created questionnaire | 30 days | OHC |
| **5. Bankruptcy** |  |  |  |  |
| Himmelstein 2014 | Direct payments | Author-created questionnaire | 2 years | OHC |
| **6. Self-reported food insecurity** | |  |  | |
| Muirhead 2009 | Cash or credit | Food insecurity questionnaire and author-created questionnaire | 12 months | OHC |
| Peltz 2019 | Direct payments | Food insecurity questionnaire and healthcare spending survey (*MEPS*) | 12 months | OHC |
| Lee 2022 | Direct payments | Food insecurity questionnaire and healthcare spending survey (*MEPS*) | 12 months | OHC |
| **7. Financial hardship experience from paying OOP for OHC** | | |  | |
| Peck 2006 | Direct payments | NA | NA | OHC |
| MacEntee 2012 | Direct payments | NA | NA | OHC |

*Studies from the WHO Regional Office for Europe. NA, Not applicable; MEPS, US Medical Expenditure Panel Survey; OOP, out-of-pocket; OHC, oral health care

**Appendix Table 5**: Methodologies to assess financial hardship used in the oral health care (OHC) context and references

| **Measures/ methods** | **Approach to analysis** | **References** |
| --- | --- | --- |
| ***1. Catastrophic spending*** | |  |
| ***1.1 Catastrophic health expenditure (CHE)*** | |  |
| *a. Budget share (household’s finances defined as)* | |  |
| - Consumption *(n=2)*   - CHE thresholds: 10%*, 25%, 40% | 1. Proportion of CHE and any OHC OOP spending 2. Share of OHC OOP spending from health care OOP spending among CHE cases | (13,35) |
|  |  |  |
| - Income *(n=2)*    - CHE thresholds: 5%, 10%* | 1. Proportion of CHE and any OHC OOP spending | (4,22) |
|  |  |  |
| *b.1 Capacity to pay (household finances = consumption minus)* | |  |
| - Partial normative food spending^a^ *(n=13)*   - CHE thresholds: 25%, 40%* | 1. Proportion of CHE and any OHC OOP spending 2. Share of OHC OOP spending from health care OOP spending among CHE cases 3. Probability of CHE and any OHC OOP spending | (1–3,15,28–32,34,36,37,41,43) |
|  |  |  |
| - Normative food, housing and utilities spending^b^ *(n=21)*   - CHE threshold: 40% | 1. Share of OHC OOP spending from health care OOP spending among CHE cases 2. Correlation between proportion of households with CHE and OHC OOP to total OOP spending ratio | (5–12,14,16–21,23,26,27,39,40,42) |
|  |  |  |
| - Total food spending^c^ *(n=2)*   - CHE threshold: 40% | 1. Proportion of CHE and any OHC OOP spending 2. Probability of CHE and any OHC OOP spending | (25,38) |
|  |  |  |
| *b.2 Capacity to pay (household finances = income minus)* | |  |
| - Partial normative food spending^a^ *(n=2)*   - CHE threshold: 40% | 1. Proportion of CHE and any OHC OOP spending 2. Probability of CHE and any OHC OOP spending | (24,33) |
|  |  |  |
| ***1.2 Catastrophic dental health expenditure (CDHE)*** | |  |
| *a. Budget share (household’s finances defined as)* | |  |
| - Consumption *(n=1)*   - CDHE threshold: 20% | 1. Proportion of CDHE | (51) |
|  |  |  |
| - Income *(n=7)*   - CDHE thresholds: 10%*, 20%, 30%, 40% | 1. Proportion of CDHE 2. Average extent exceeded from the catastrophic threshold (overshoot) 3. Mean overshoot among CDHE cases 4. Proportion of CDHE among CHE cases | (45–47,49,50,52) |
|  |  |  |
| *b. Capacity to pay (household finances = consumption minus)* | |  |
| - Total food spending^c^ *(n=1)*   - CDHE thresholds: 30% | 1. Proportion of CDHE | (48) |
|  |  |  |
| - Partial normative food spending^a^ *(n=1)*   - CDHE threshold: 40% | 1. Proportion of CDHE | (53) |
|  |  |  |
| ***2. Impoverishment (IMPOV)*** |  |  |
| - Household finances= consumption *(n=1)*   - Poverty line: 44th and 55th ranked household food consumption average from the sample | 1. Proportion of IMPOV and any OHC OOP spending 2. Probability of IMPOV and OHC OOP spending | (43) |
| ***3. Self-reported financial burden from OHC*** | |  |
| - “In the past 3 years has the cost of dental been a financial burden to you?” *(n=1)*    - Indicator: “Yes” | 1. Proportion of self-reported financial burden from OHC | (54) |
|  |  |  |
| - “In the last 12 months, how much of a financial burden have dental visits been for you?” *(n=2)*   - Indicator: “A large burden” | 1. Proportion of self-reported financial burden from OHC | (55,56) |
|  |  |  |
| - “Does your dental expenditure affect other day to day expenditure?” *(n=1)*    - Indicator: “Yes” | 1. Proportion of self-reported financial burden from OHC | (52) |
|  |  |  |
| - “Do you feel dental expenditure is really a burden to your family?” *(n=1)*   - Indicator: “Yes” | 1. Proportion of self-reported financial burden from OHC | (52) |
|  |  |  |
| - “To what extent were the costs of dental examinations or treatments a financial burden to your household during the past 12 months?” *(n=1)*    - Indicator: “A heavy burden” or “somewhat a burden” | 1. Proportion of self-reported financial burden from OHC | (58) |
|  |  |  |
| - Financial impact on dental caries treatment*^ø^ (n=1)*    - Indicator: “Serious impact” or “very serious impact” | 1. Proportion of self-reported financial burden from OHC | (57) |
|  |  |  |
| ***4. Distress financing*** |  |  |
| - Type of financing households used to pay for health care*^ø^* *(n=1)*   - Indicator: (Any of the following) loans; selling assets; extra working hours; ex-gratia payment by other household member; reducing food spending; removing children from school | 1. Proportion of distress financing for OHC OOP spending | (59) |
| ***5. Bankruptcy*** |  |  |
| - Unable to keep up with their debts and have either to sell all of their assets or reach an agreement with their debtors *(n=1)*   - Indicator: Insolvency files | 1. Proportion of participants experiencing bankruptcy and spending a “large amount” OOP on OHC | (60) |
|  |  |  |
| ***6. Self-reported food insecurity*** |  |  |
| - Presence of food insecurity issues (8 or 10 questions)*^†^ (n=2)* - Indicator: “Yes” to any food insecurity issue | 1. Proportion of participants self-reporting food insecurity and any OHC OOP spending 2. Probability of experiencing food insecurity and any OHC OOP spending | (62,63) |
|  |  |  |
| - Frequency of food insecurity issues (3 questions) in terms of being worried there would be enough to eat in the last year, did not have enough to eat, or did not eat the desired quality or variety of food because of lack of money*^¥^ (n=1)*    - Indicator: “Often” or “sometimes” to any food insecurity issue |  | (61) |
|  |  |  |
| ***7. Financial hardship experience from paying OOP for OHC*** | |  |
| - In depth interview on the “sequential costs” (i.e., risking paying rent) due to OHC OOP spending *(n=1)* | 1. Thematic analysis | (64) |
|  |  |  |
| - Focus group on accessing OHC *(n=1)* | 1. Narrative analysis | (65) |

**Indicates the most frequent threshold used among reviewed studies. OOP, out-of-pocket; OHC, oral health care.*

*^a^Calculated by averaging the 44th and 55th ranked household food consumption from the sample (among those with a negative capacity to pay the actual food spending is employed). ^b^Calculated by averaging the food, rent, and utilities (water, electricity, gas and other fuels used for cooking and heating) spending from the 25th and 35th ranked household consumption (equivalized to household size and composition) from the sample (those with a negative capacity to pay are considered catastrophic). ^c^Calculated from the household’s total food consumption.* *^ø^Exact phrasing of the question is unavailable; ^†^From the 2016/17 Medical Expenditure Panel Survey in the USA; ^¥^From the 2003 Canadian Community Health Survey*

**References for supplementary Appendix Table 2 and 4**

1. World Health Organization, Mathauer I, Xu K, Carrin G, Evans DB. An analysis of the health financing system of the Republic of Korea and options to strengthen health financing performance. Geneva: World Health Organization; 2009.

2. World Health Organization. Financial burden of health payments in France: 1995-2006. Geneva: World Health Organization; 2010.

3. Kim Y, Yang B. Relationship between catastrophic health expenditures and household incomes and expenditure patterns in South Korea. Health Policy. 2011 May;100(2–3):239–46.

4. Willink A, Schoen C, Davis K. Dental Care And Medicare Beneficiaries: Access Gaps, Cost Burdens, And Policy Options. Health Aff Proj Hope. 2016 Dec 1;35(12):2241–8.

5. Cooke O’Dowd N, Kumpunen S, Holder H. Can people afford to pay for health care? New evidence on financial protection in the United Kingdom. Copenhagen: World Health Organization. Regional Office for Europe; 2018.

6. Czypionka T, Röhrling G, Six E. Can people afford to pay for health care? New evidence on financial protection in Austria. Copenhagen: World Health Organization. Regional Office for Europe; 2018.

7. Murauskienė L, Thomson S. Can people afford to pay for health care? New evidence on financial protection in Lithuania. Copenhagen: World Health Organization. Regional Office for Europe; 2018.

8. Siegel M, Busse R. Can people afford to pay for health care? New evidence on financial protection in Germany. Copenhagen: World Health Organization. Regional Office for Europe; 2018.

9. Vončina L, Rubil I. Can people afford to pay for health care? New evidence on financial protection in Croatia. Copenhagen: World Health Organization. Regional Office for Europe; 2018.

10. Võrk A, Habicht T. Can people afford to pay for health care? New evidence on financial protection in Estonia. Copenhagen: World Health Organization. Regional Office for Europe; 2018.

11. Taube M, Vaskis E, Nesterenko O. Can people afford to pay for health care? New evidence on financial protection in Latvia. Copenhagen: World Health Organization. Regional Office for Europe; 2018.

12. Thomson S, Evetovits T, Cylus J. Financial protection in high-income countries: a comparison of the Czech Republic, Estonia and Latvia. Copenhagen: World Health Organization. Regional Office for Europe; 2018.

13. Edmonds S, Hajizadeh M. Assessing progressivity and catastrophic effect of out-of-pocket payments for healthcare in Canada: 2010–2015. Eur J Health Econ. 2019 Sep;20(7):1001–11.

14. Glenngård AH, Borg S. Can people afford to pay for health care? New evidence on financial protection in Sweden: summary. Copenhagen: World Health Organization. Regional Office for Europe; 2019.

15. Quintal C, Lopes J. Catastrophic Expenditure on Medicines: An Analysis Based on the Portuguese Household Budget Survey 2015/2016. In 2019. p. 7269–75.

16. Johnston B, Thomas S, Burke S. Can people afford to pay for health care? New evidence on financial protection in Ireland. Copenhagen: World Health Organization. Regional Office for Europe; 2020.

17. Tambor M, Pavlova M. Can people afford to pay for health care? New evidence on financial protection in Poland. Copenhagen: World Health Organization. Regional Office for Europe; 2020.

18. Kontemeniotis A, Theodorou M. Can people afford to pay for health care? New evidence on financial protection in Cyprus. Copenhagen: World Health Organization. Regional Office for Europe; 2021.

19. Tervola J, Aaltonen K, Tallgren F. Can people afford to pay for health care? New evidence on financial protection in Finland. Copenhagen: World Health Organization. Regional Office for Europe; 2021.

20. Urbanos-Garrido RM, Peña-Longobardo LM, Comendeiro-Maaløe M, Oliva J, Ridao-López M, Bernal-Delgado E. Can people afford to pay for health care? New evidence on financial protection in Spain. Copenhagen: World Health Organization. Regional Office for Europe; 2021.

21. Gabriela Scîntee S, Mosca I, Vlădescu C. Can people afford to pay for health care? New evidence on financial protection in Romania. Copenhagen: World Health Organization. Regional Office for Europe; 2022.

22. Liu C, Scannell CA, Kenison T, Wren SM, Saliba D. Improvements and Gaps in Financial Risk Protection Among Veterans Following the Affordable Care Act. J Gen Intern Med. 2022 Feb;37(3):573–81.

23. Can people afford to pay for health care? new evidence on financial protection in Belgium. Copenhagen: WHO Regional Office for Europe; 2023.

24. Shokri A, Bolbanabad AM, Rezaei S, Moradi G, Piroozi B. Has Iran achieved the goal of reducing the prevalence of households faced with catastrophic health expenditure to 1%?: A national survey. Health Sci Rep. 2023;6(4).

25. Proaño Falconi D, Bernabé E. Determinants of catastrophic healthcare expenditure in Peru. Int J Health Econ Manag. 2018 Dec;18(4):425–36.

26. Garam I Zadnipru, Mariana, Doronin, Valeriu, Matei, Andrei and Ilaria Mosca. Can people afford to pay for health care? New evidence on financial protection in the Republic of Moldova. Copenhagen: World Health Organization. Regional Office for Europe; 2020.

27. Tomini F, Tomini S M. Can people afford to pay for health care?: new evidence on financial protection in Albania: summary. Copenhagen: World Health Organization. Regional Office for Europe; 2020.

28. Kavosi Z, Rashidian A, Pourreza A, Majdzadeh R, Pourmalek F, Hosseinpour AR, et al. Inequality in household catastrophic health care expenditure in a low-income society of Iran. Health Policy Plan. 2012;27(7):613–23.

29. Kavosi Z, Keshtkaran A, Hayati R, Ravangard R, Khammarnia M. Household financial contribution to the health System in Shiraz, Iran in 2012. Int J Health Policy Manag. 2014 Oct;3(5):243–9.

30. Moradi G, Safari H, Piroozi B, Qanbari L, Farshadi S, Qasri H, et al. Catastrophic health expenditure among households with members with special diseases: A case study in Kurdistan. Med J Islam Repub Iran. 2017;31:43.

31. Mehdizadeh P, Daniyali H, Meskarpour-Amiri M, Dopeykar N, Uzi H. Catastrophic and impoverishing health expenditures and it’s affecting factors among health staffs in Iran: A case study in Tehran. Med J Islam Repub Iran. 2019;33(8910777):120.

32. Nemati E, Khezri A, Nosratnejad S. The Study of Out-of-pocket Payment and the Exposure of Households with Catastrophic Health Expenditures Following the Health Transformation Plan in Iran. Risk Manag Healthc Policy. 2020;13:1677–85.

33. Khammarnia M, Setoodehzadeh F, Ansari-Moghaddam A, Barfar E, Zanganeh Baygi M, Peyvand M. Household financial contribution to the health system after Iran’s Health Transformation Plan. Rural Remote Health. 2020 Feb;20(1):5495.

34. Ahmadi R, Shafiei M, Ameri H, Askari R, Fallahzadeh H. Catastrophic Health Expenditure before and after of the Implementation of Health Sector Evolution Plan in Iran. Inq J Med Care Organ Provis Financ. 2021 Dec;58:469580211050210.

35. Hsu J, Majdzadeh R, Mills A, Hanson K. A dominance approach to analyze the incidence of catastrophic health expenditures in Iran. Soc Sci Med 1982. 2021 Sep;285:114022.

36. Barfar E, Pourreza A, Sharifi V, Sobhanian SMH, Akbari Sari A. Catastrophic Health Expenditure in Households with Severe Mental Disorders Patients: Evidence After Iran’s Health Transformation Plan. Health Scope. 2019 May 1;In Press(In Press).

37. Ghorbanian A, Rashidian A, Lankarani K, Kavosi Z. The Prevalence and Determinants of Catastrophic Health Expenditures in Iran: A Systematic Review and Meta-Analysis. Health SCOPE. 2019;8(1).

38. Aeenparast A, Bayati M, Frzadi F, Haeri Mehrizi AA, Ayoubian A. Estimating the Catastrophic Health Cost in Iranian Health Insurance Policyholders and Its Relevant Factors. Health Educ Health Promot. 2022;10(3).

39. Goroshko A, Shapoval N, Lai T. Can people afford to pay for health care? New evidence on financial protection in Ukraine. Copenhagen: World Health Organization. Regional Office for Europe; 2018.

40. Jakab M, Akkazieva B, Habicht J. Can people afford to pay for health care? New evidence on financial protection in Kyrgyzstan. Copenhagen: World Health Organization. Regional Office for Europe; 2018.

41. Woldemichael A, Rezaei S, Kazemi Karyani A, Ebrahimi M, Soltani S, Aghaei A. The impact of out-of pocket payments of households for dental healthcare services on catastrophic healthcare expenditure in Iran. BMC Public Health. 2021 Jul 28;21(1):1474.

42. Thomson S, Cylus J, Evetovits Tamás. Can people afford to pay for health care? new evidence on financial protection in Europe: [regional report]. Copenhagen: World Health Organization, Regional Office for Europe; 2019.

43. Bernabé E, Masood M, Vujicic M. The impact of out-of-pocket payments for dental care on household finances in low and middle income countries. BMC Public Health. 2017 Jan 23;17:109.

44. López-López S, Del Pozo-Rubio R, Ortega-Ortega M, Escribano-Sotos F. Catastrophic out-of-pocket payments for dental treatment: regional evidence from Spain. BMC Health Serv Res. 2023 Jul 22;23(1):784.

45. Snow P., McNally M.E. Examining the implications of dental treatment costs for low-income families. J Can Dent Assoc. 2010;76((Snow) Dalhousie University, Halifax, Nova Scotia.):a28.

46. AlBaty A, AlGhasham H, Al Wusaybie M, El Tantawi M. Dental expenditure and catastrophic dental expenditure in Eastern Saudi Arabia: Pattern and associated factors. J Clin Exp Dent. 2019 Jul;11(7):e601–8.

47. López-López S, Del Pozo-Rubio R, Ortega-Ortega M, Escribano-Sotos F. Catastrophic household expenditure associated with out-of-pocket payments for dental healthcare in Spain. Eur J Health Econ HEPAC Health Econ Prev Care. 2022 Sep;23(7):1187–201.

48. Pérez-Núñez R, Vargas-Palacios A, Ochoa-Moreno I, Medina-Solis CE. Household expenditure in dental health care: national estimations in Mexico for 2000, 2002, and 2004. J Public Health Dent. 2007 Fall;67(4):234–42.

49. Sun X, Bernabé E, Liu X, Gallagher JE, Zheng S. Determinants of Catastrophic Dental Health Expenditure in China. PloS One. 2016;11(12):e0168341.

50. Nyamuryekung’e KK, Lahti S, Tuominen R. Costs of dental care and its financial impacts on patients in a population with low availability of services. Community Dent Health. 2019;36(2):131–6.

51. Prasad BM, Tripathy JP, Bera OP, Shanbhag N. National sample surveys show poor households face catastrophic expenditure for oral healthcare services in India. J Fam Med Prim Care. 2021 Aug;10(8):2853–8.

52. Nobelika A, Simon A. An Observational Study to find the Patterns of Out-of-Pocket Expenditure for Oral Healthcare among Sanitary Workers in Coimbatore, India. J INDIAN Assoc PUBLIC Health Dent. 2020;18(2):143–50.

53. Masood M, Sheiham A, Bernabé E. Household expenditure for dental care in low and middle income countries. PloS One. 2015;10(4):e0123075.

54. Locker D, Maggirias J, Quiñonez C. Income, dental insurance coverage, and financial barriers to dental care among Canadian adults: Financial barriers to dental care. J Public Health Dent. 2011 Sep;71(4):327–34.

55. Chrisopoulos S, Harford J. Oral health and dental care in Australia: key facts and figures 2012. Canberra: AIHW; 2013.

56. Australian Research Centre for Population Oral Health. Financial burden of dental care among Australian children. Aust Dent J. 2014 Jun;59(2):268–72.

57. Uguru N, Onwujekwe O, Uguru CC, Ogu UU. Achieving universal health coverage in Nigeria: the dilemma of accessing dental care in Enugu state, Nigeria, a mixed methods study. Heliyon. 2021 Jan;7(1):e05977.

58. Vojvodic K, Terzic-Supic Z, Todorovic J, Gagliardi C, Santric-Milicevic M, Popovic M. Financial Burden of Medical Care, Dental Care, and Medicines among Older-Aged Population in Slovenia, Serbia, and Croatia. Int J Environ Res Public Health. 2022 Mar 11;19(6):3325.

59. Rahman MM, Gilmour S, Saito E, Sultana P, Shibuya K. Self-reported illness and household strategies for coping with health-care payments in Bangladesh. Bull World Health Organ. 2013;91(6):449–58.

60. Himmelstein DU, Woolhandler S, Sarra J, Guyatt G. Health issues and health care expenses in Canadian bankruptcies and insolvencies. Int J Health Serv Plan Adm Eval. 2014;44(1):7–23.

61. Muirhead V, Quiñonez C, Figueiredo R, Locker D. Oral health disparities and food insecurity in working poor Canadians. Community Dent Oral Epidemiol. 2009 Aug;37(4):294–304.

62. Peltz A, Garg A. Food insecurity and health care use. Pediatrics. 2019;144(4).

63. Lee WC, Lin S, Yang TC, Serag H. Cross-sectional study of food insecurity and medical expenditures by race and ethnicity. Ethn Health. 2022;

64. Peck LR, Segal EA. The latent and sequential costs of being poor: Exploration of a potential paradigm shift. J Poverty. 2006;10(1):1–24.

65. MacEntee M.I., Marino R., Wong S., Kiyak A., Minichiello V., Chi I., et al. Discussions on oral health care among elderly Chinese immigrants in Melbourne and Vancouver. Gerodontology. 2012;29(2):e822-832.

**Appendix Table 6**: Measurement challenges in studies included

| **Measure/studies** | **Measurement challenges** | |
| --- | --- | --- |
|  | **Reported challenge** | **Type of challenge** |
| **1. Catastrophic spending**  **1.1 Catastrophic health expenditure** | | |
| WHO 2009 | NONE REPORTED | - |
| WHO 2010 | NONE REPORTED | - |
| Kim 2011 | NONE REPORTED | - |
| Kavosi 2012 | 1) Low-income who forgo their needs and do not consume health care are left out of CHE calculations. | Conceptual |
|  | 2) Sample size, wide confidence intervals | Design |
|  | 3) Recall bias | Instrument |
|  | 4) Limited to one district in Tehran affects generalizability | Design |
| Kavosi 2014 | 1) Over or under reporting of the costs | Instrument |
|  | 2) Recall bias | Instrument |
| Willink 2016 | 1) No independent source to verify respondents' reported use or expenses on OHC | Instrument |
| Bernabé 2017 | 1) World Health Survey data are over a decade old | Instrument |
|  | 2) Cross-sectional data | Design |
|  | 4) Overlap between 8 types of health services | Instrument |
| Moradi 2017 | 1) Not representative | Design |
|  | 2) Over or under reporting of the expenditures | Instrument |
|  | 3) Recall bias | Instrument |
|  | 4) No information on money saving or borrowing | Instrument |
| Cooke O’Dowd 2018 | 1) Financial hardship does not capture financial barrier | Conceptual |
| Czypionka 2018 | 1) Financial hardship does not capture financial barrier | Conceptual |
| Goroshko 2018 | 1) Financial hardship does not capture financial barrier | Conceptual |
| Jakab 2018 | 1) Financial hardship does not capture financial barrier | Conceptual |
| Murauskienė 2018 | 1) Financial hardship does not capture financial barrier | Conceptual |
| Proaño Falconí 2018 | 1) Cross-sectional data | Design |
|  | 2) Excluded households due to missing value from household members | Instrument |
|  | 3) Study sample included lower income and smaller households and those where all members have health insurance– not necessarily generalizable | Design |
|  | 4) Different recall periods among the healthcare services | Instrument |
|  | 5) Indirect costs (transportation and missing work hours) not collected | Instrument |
| Siegel 2018 | 1) Financial hardship does not capture financial barrier | Conceptual |
| Taube 2018 | 1) Financial hardship does not capture financial barrier | Conceptual |
| Thomson 2018 | 1) Financial hardship does not capture financial barrier | Conceptual |
| Vončina 2018 | 1) Financial hardship does not capture financial barrier | Conceptual |
| Võrk 2018 | 1) Financial hardship does not capture financial barrier | Conceptual |
| Barfar 2019 | NONE REPORTED | - |
| Edmonds 2019 | NONE REPORTED | - |
| Ghorbanian 2019 | 1) Cross-sectional design | Instrument |
|  | 2) Close-ended questions | Instrument |
|  | 3) Not generalizable | Design |
|  | 4) Reporting error | Instrument |
|  | 5) Social desirability/participant bias | Instrument |
|  | 6) OOP payments did not capture traditional or home care remedies | Instrument |
| Glenngård 2019 | 1) Financial hardship does not capture financial barrier | Conceptual |
| Mehdizadeh 2019 | 1) Recall bias | Instrument |
|  | 2) Framing of the expenditure questions | Instrument |
| Quintal 2019 | NONE REPORTED | - |
| Thomson 2019 | 1) Financial hardship does not capture financial barrier | Conceptual |
| Garam 2020 | 1) Financial hardship does not capture financial barrier | Conceptual |
| Johnston 2020 | 1) Financial hardship does not capture financial barrier | Conceptual |
| Khammarnia 2020 | 1) Not nationally representative | Design |
|  | 2) Cross-sectional design | Design |
|  | 3) Over or underreporting of costs | Instrument |
|  | 4) Recall bias | Instrument |
| Nemati 2020 | NONE REPORTED | - |
| Tambor 2020 | 1) Financial hardship does not capture financial barrier | Conceptual |
| Tomini 2020 | 1) Financial hardship does not capture financial barrier. | Conceptual |
| Ahmadi 2021 | 1) The missed and replaced households were not exposed to CHE | Design |
|  |  |  |
| Hsu 2021 | 1) Survey structure modified in 2016 by merging similar categories of medical expenditure, reducing the number of questions and distinguishing spending by provider setting – estimations of OOP payers are sensitive to the number of questions. | Instrument |
| Kontemeniotis 2021 | 1) Financial hardship does not capture financial barrier | Conceptual |
| Tervola 2021 | 1) Financial hardship does not capture financial barrier | Conceptual |
| Urbanos-Garrido 2021 | 1) Financial hardship does not capture financial barrier | Conceptual |
| Woldemichael 2021 | 1) Cross sectional data | Design |
|  | 2) Measuring underlying catastrophic health expenditure factors | Instrument |
| Aeenparast 2022 | NONE REPORTED | - |
| Liu 2022 | 1) Unable to identify veterans with forms of military discharge other than honorable, who may experience greater financial risk due to ineligibility for veterans’ benefits | Design |
|  | 2) Survey follows respondents for at most 2 years, preventing longitudinal analysis of individual veterans whose insurance coverage changed | Instrument |
|  | 3) Broader economic trends in the US, including gains in employment and income, may affect results | Conceptual |
|  | 4) Residual confounding from redistribution of veterans to lower-cost areas within census regions during the study period | Design |
|  | 5) Spillover effects of the non-veteran family members | Design |
|  | 6) Survey does not include medical debt and might underestimate some veterans’ financial hardship | Instrument |
| Scîntee 2022 | 1) Financial hardship does not capture financial barrier | Conceptual |
| Boukaert, 2023 | 1) Financial hardship does not capture financial barrier | Conceptual |
| Shokri, 2023 | 1) Recall bias | Instrument |
| **1.2 Catastrophic dental health expenditure** | |  |
| Pérez-Nuñez 2007 | 1) Spending on medication was not included | Instrument |
|  | 2) Can't know who needed but did not use OHC | Instrument |
|  | 3) Not possible to know if a households received free OHC (no information on utilization) | Instrument |
| Snow 2010 | 1) Income was assumed for the scenarios and not recorded by patients. | Instrument |
|  | 2) Patient’s ability to complete treatment might have been limited by the amount of work that could be completed within the 1-year period | Design |
| Masood 2015 | 1) Recall bias | Instrument |
|  | 2) No information on indirect costs (i.e., income loss due to ill health, travel, waiting at health care facilities or providing care to family members) | Instrument |
|  | 3) Analysis did not allow the assessment of the cumulative effect of oral diseases and recurrent restorative treatment on expenditure on OHC | Instrument |
|  | 4) Estimates of heath spending are higher when using more health expenditure questions and lower when using more non-health expenditure questions and longer recall periods | Instrument |
| Sun 2016 | 1) Cross-sectional design | Design |
|  | 2) Old dataset (2005) | Design |
|  | 3) Recall period may be too long (12 months) | Instrument |
|  | 4) Using income in the denominator may not be responsive to the means of financing health care (savings, loans, selling assets, income transfers). | Instrument |
|  | 5) Does not capture those who could not afford OHC | Conceptual |
| Nyamuryekung'e 2019 | 1) Informal employment impact income stability and produce frequent fluctuations | Conceptual |
|  | 2) Many of the population are subsistence farmers who consume the food they produce | Conceptual |
|  | 3) Difficulty measuring household consumption | Instrument |
|  | 4) Response rate for open ended household income was lower than a category response | Instrument |
|  | 5) Monthly income may underestimate the overall household consumption | Instrument |
|  | 6) The household financial impact experienced will vary depending on the household member seeking treatment, since the impact is greater on the main earners | Instrument |
|  | 7) Non-inclusion of medication fees in the cost calculation (those prescribed or self-medicated) | Instrument |
| AlBaty 2019 | 1) Convenience sample | Design |
|  | 2) Recall bias | Instrument |
| Nobelika 2020 | 1) Cross-sectional design | Design |
|  | 2) Close-ended questions | Instrument |
|  | 3) Generalizability | Design |
|  | 4) Reporting error. Self‑reporting of income and OOP payments for OHC may be an overlap of expenditure. | Instrument |
|  | 5) Presence of social desirability/participant bias. | Design |
|  | 6) OOP payments did not capture traditional or home care remedies. | Instrument |
| Prasad 2021 | 1) No information on the type of oral healthcare services provided | Instrument |
|  | 2) Indirect costs due to loss of wage were not considered in this study | Design |
|  | 3) Recall bias | Instrument |
| López-López 2022 | 1) No panel dataset | Design |
|  | 2) Lack of information on the type and /or severity of the OHC related problem, the duration of treatment or the drugs or prosthetics prescribed | Instrument |
|  | 3) Lack of information on private health insurance for households | Instrument |
| López-López 2023 | 1) No longitudinal/panel dataset | Design |
|  | 2) Old dataset | Instrument |
| **2. Impoverishment** | |  |
| Bernabé 2017 | 1) World Health Survey data are over a decade old | Instrument |
|  | 2) Cross-sectional data | Design |
|  | 3) Expenditure estimates were derived from 14 items with a recall frame of 4 weeks | Instrument |
|  | 4) Overlap between 8 types of health services | Instrument |
| **3. Self-reported financial burden from OHC** | |  |
| Locker 2011 | NONE REPORTED | - |
| Chrisopoulos 2013 | NONE REPORTED | - |
| ARCPOH 2014 | NONE REPORTED | - |
| Nobelika 2020 | 1) Cross-sectional design | Design |
|  | 2) Close-ended questions | Instrument |
|  | 3) Generalizability | Design |
|  | 4) Reporting error. Self‑reporting of income and OOP payments for OHC may be an overlap of expenditure. | Instrument |
|  | 5) Presence of social desirability/participant bias. | Design |
|  | 6) OOP payments did not capture traditional or home care remedies. | Instrument |
| Uguru 2021 | NONE REPORTED | - |
| Vojvodic 2022 | 1) Recall bias and underreporting | Instrument |
|  | 2) Exclude older aged people living in collectives | Design |
|  | 3) Not possible to conclude which family member generated most dental costs | Instrument |
|  | 4) Excludes compulsory or voluntary health insurance | Design |
|  | 5) Private practice for OHC was not the subject of the study | Conceptual |
| **4. Distress financing** |  |  |
| Rahman 2013 | 1) Only measured urban households in a single metropolitan area of Bangladesh | Design |
|  | 2) Episodes of illness were self-reported | Instrument |
|  | 3) Did not estimate indirect costs | Instrument |
| **5. Bankruptcy** |  |  |
| Himmelstein 2014 | 1) Low response rate | Instrument |
|  | 2) Debtors with medical problems were more likely to respond | Design |
|  | 3) Verification on the accuracy of responses | Instrument |
| **6. Self-reported food insecurity** | |  |
| Muirhead 2009 | 1) Study restricted to landline telephones users - sampling bias | Design |
|  | 2) Simplified "red flag" food insecurity approach may lack precision compared to other comprehensive food insecurity questionnaires | Instrument |
| Peltz 2019 | NONE REPORTED | - |
| Lee 2022 | 1) Neighbourhood characteristics were not analyzed | Design |
|  | 2) Can’t measure causal relationship | Design |
|  | 3) Definition of food insecurity may differ in different governmental institutions | Instrument |
| **7. Financial hardship experience from paying OOP for OHC** | |  |
| Peck 2006 | NONE | - |
| MacEntee 2012 | 1) Difficulty in recruiting immigrants with low income/education or those who felt socially isolated. | Design |
|  | 2) Lack of information on personal income | Instrument |
|  | 3) Recruitment strategies to involve immigrants who are socially disengaged to those experiencing financial problems may be different | Design |

WHO, World Health Organization; OOP, out-of-pocket; OHC, oral health care.**Appendix Table 7**: Evidence gaps of financial hardship in the oral health care context in studies included

| **Measure/studies** | **Evidence gaps** |
| --- | --- |
| **1. Catastrophic spending**  **1.1 Catastrophic health expenditure** | |
| WHO 2009 | NONE REPORTED |
| WHO 2010 | NONE REPORTED |
| Kim 2011 | NONE REPORTED |
| Kavosi 2012 | 1) Estimate the bias introduced when using expenditure data |
|  | 2) Incorporate the unmet needs of low-income households in the analysis |
|  | 3) Assess the impacts of different interventions on catastrophic health expenditure level |
| Kavosi 2014 | 1) Examine the behavior of households suffering from serious medical conditions |
| Willink 2016 | 1) Verify respondents' reported use or expenses on OHC |
| Bernabé 2017 | 1) Explore mechanisms families use to cope with OOP expenditures for OHC |
|  | 2) Evaluate the impact of specific dental treatments, disease treatment vs cosmetic/aesthetic treatment |
| Moradi 2017 | NONE REPORTED |
| Cooke O’Dowd 2018 | NONE REPORTED |
| Czypionka 2018 | NONE REPORTED |
| Goroshko 2018 | NONE REPORTED |
| Jakab 2018 | NONE REPORTED |
| Murauskienė 2018 | NONE REPORTED |
| Proaño Falconí 2018 | 1) Assess the long-term financial effect of OOP payments for healthcare services |
|  | 2) Assess the role of multiple health shocks in treating chronic conditions |
|  | 3) Assess the repeated utilization of healthcare services to treat chronic conditions |
| Siegel 2018 | NONE REPORTED |
| Taube 2018 | NONE REPORTED |
| Thomson 2018 | NONE REPORTED |
| Vončina 2018 | NONE REPORTED |
| Võrk 2018 | NONE REPORTED |
| Barfar 2019 | NONE REPORTED |
| Edmonds 2019 | 1) Expand the study to cover the territories. |
| Ghorbanian 2019 | NONE REPORTED |
| Glenngård 2019 | NONE REPORTED |
| Mehdizadeh 2019 | NONE REPORTED |
| Quintal 2019 | NONE REPORTED |
| Thomson 2019 | NONE REPORTED |
| Garam 2020 | NONE REPORTED |
| Johnston 2020 | NONE REPORTED |
| Khammarnia 2020 | 1) Investigate the effects of national health insurance interventions |
| Nemati 2020 | NONE REPORTED |
| Tambor 2020 | NONE REPORTED |
| Tomini 2020 | 1) Capture financial barriers to access that result in unmet need for health care |
| Ahmadi 2021 | NONE REPORTED |
| Hsu 2021 | 1) Effect of sanctions on Iran with causal inference |
|  | 2) Examination of alternative statistical tests for dominance |
| Kontemeniotis 2021 | NONE REPORTED |
| Tervola 2021 | NONE REPORTED |
| Urbanos-Garrido 2021 | NONE REPORTED |
| Woldemichael 2021 | 1) Identify factors contributing to the increased probability of catastrophic health expenditure |
| Aeenparast 2022 | 1) Investigate the effect of health policies on catastrophic health expenditure in Iran |
| Liu 2022 | NONE REPORTED |
| Scîntee 2022 | NONE REPORTED |
| Boukaert, 2023 | NONE REPORTED |
| Shokri, 2023 | NONE REPORTED |
| **1.2 Catastrophic dental health expenditure** | |
| Pérez-Núñez 2007 | 1) Compare between regions/states within Mexico |
| Snow 2010 | 1) Affordability of OHC for the patient population of the Dalhousie dental clinic |
| Masood 2015 | 1) Mechanisms families use to cope with OOP payments |
|  | 2) The specific role of health and dental health insurance in reducing catastrophic dental health expenditure |
|  | 3) What specific dental service may force families into catastrophic payment |
| Sun 2016 | 1) Evaluation of the impact of health insurance expansion experience in China over the last decade on catastrophic dental health expenditure |
|  | 2) Consider the impact of specific dental treatments, particularly, those that are considered essential (disease treatment) and cosmetic. |
| Nyamuryekung'e 2019 | NONE REPORTED |
| AlBaty 2019 | NONE REPORTED |
| Nobelika 2020 | NONE REPORTED |
| Prasad 2021 | NONE REPORTED |
| López-López 2022 | 1) Estimate the effect of treating secondary pathologies attributed to primary oral dental pathologies that are currently untreated due to the lack of public health coverage. |
| López-López 2023 | 1) Quantifying the level of OOP spending per household and by region, on OHC |
|  | 2) Estimate the total costs of OHC needs |
|  | 3) Estimate the secondary effect of dental pathologies on other health care needs |
|  | 4) Understand the proportion of catastrophic dental health expenditure by relevant sociodemographic factors (sex, education, financial status, and place of residence) in regions of Spain |
| **2. Impoverishment** | |
| Bernabé 2017 | 1) Explore mechanisms families use to cope with OOP expenditures for OHC |
|  | 2) Evaluate the impact of specific dental treatments, particularly those that are essential (disease treatment) and cosmetic/aesthetic |
| **3. Self-reported financial burden from OHC** | |
| Locker 2011 | NONE REPORTED |
| Chrisopoulos 2013 | NONE REPORTED |
| ARCPOH 2014 | NONE REPORTED |
| Nobelika 2020 | NONE REPORTED |
| Uguru 2021 | NONE REPORTED |
| Vojvodic 2022 | 1) Understand the structure of healthcare financial hardship and its causality considering socio-economic characteristics, health insurance and health systems characteristics |
| **4. Distress financing** |  |
| Rahman 2013 | NONE REPORTED |
| **5. Bankruptcy** |  |
| Himmelstein 2014 | 1) Longitudinal designs |
| **6. Self-reported food insecurity** | |
| Muirhead 2009 | 1) Assess the full extent of food insecurity |
|  | 2) Employ life-course and multidisciplinary approaches to understand the complex causal pathways and decision-making processes related to food insecurity and oral ill-health |
| Peltz 2019 | NONE REPORTED |
| Lee 2022 | 1) Need for studies to distinguish among the Hispanic population |
| **7. Financial hardship experienced from paying OOP for OHC** | |
| Peck 2006 | 1) Quantify costs identified in the study |
|  | 2) Develop an empirical model for presenting the latent and sequential costs proposed in the study |
|  | 3) Explore the determinants of varied types of latent and sequential costs proposed in the study |
|  | 4) Identify the extent to which experiencing varying levels of latent or sequential costs impacts the financial hardship families face |
|  | 5) Assess the population’s latent and sequential costs |
| MacEntee 2012 | NONE REPORTED |

OOP, out-of-pocket; OHC, oral health care.
